# CD70 expression determines the therapeutic efficacy of expanded human regulatory T cells

**DOI:** 10.1038/s42003-020-1097-8

**Published:** 2020-07-14

**Authors:** Rebeca Arroyo Hornero, Christos Georgiadis, Peng Hua, Dominik Trzupek, Li-Zhen He, Waseem Qasim, John A. Todd, Ricardo C. Ferreira, Kathryn J. Wood, Fadi Issa, Joanna Hester

**Affiliations:** 1 Transplantation Research and Immunology Group, Nuffield Department of Surgical Sciences, John Radcliffe Hospital, University of Oxford, Oxford, OX3 9DU UK; 2 Molecular and Cellular Immunology Unit, UCL Great Ormond Street Institute of Child Health, London, WC1N 1EH UK; 3 MRC Molecular Haematology Unit, Weatherall Institute of Molecular Medicine, Radcliffe Department of Medicine, John Radcliffe Hospital, Oxford, OX3 9DS UK; 4 JDRF/Wellcome Diabetes and Inflammation Laboratory, Wellcome Centre for Human Genetics, Nuffield Department of Medicine, NIHR Oxford Biomedical Research Centre, University of Oxford, Oxford, OX3 7BN UK; 5 Celldex Therapeutics, Inc., Hampton, NJ 08827 USA

**Keywords:** Translational immunology, Lymphocyte activation, Regulatory T cells

## Abstract

Regulatory T cells (Tregs) are critical mediators of immune homeostasis. The co-stimulatory molecule CD27 is a marker of highly suppressive Tregs, although the role of the CD27-CD70 receptor-ligand interaction in Tregs is not clear. Here we show that after prolonged in vitro stimulation, a significant proportion of human Tregs gain stable CD70 expression while losing CD27. The expression of CD70 in expanded Tregs is associated with a profound loss of regulatory function and an unusual ability to provide CD70-directed co-stimulation to TCR-activated conventional T cells. Genetic deletion of CD70 or its blockade prevents Tregs from delivering this co-stimulatory signal, thus maintaining their regulatory activity. High resolution targeted single-cell RNA sequencing of human peripheral blood confirms the presence of CD27^−^CD70^+^ Treg cells. These findings have important implications for Treg-based clinical studies where cells are expanded over extended periods in order to achieve sufficient treatment doses.

## Abstract (web summary)

Regulatory T cells maintain immune homeostasis and help control development of autoimmunity and allergy. Arroyo-Hornero et al show that upon prolonged stimulation, regulatory T cells may switch to an immunostimulatory phenotype by upregulating the expression of the co-stimulatory molecule CD70.

## Introduction

CD4^+^FOXP3^+^ regulatory T cells (Tregs) have a critical role in the maintenance of immunological self-tolerance and immune homeostasis. Tregs are also the subject of investigation as a cellular therapy for the therapeutic induction of tolerance to alloantigens or autoantigens^1–3^. Treg function is dependent on their stable expression of the transcription factor FOXP3^4^, largely determined by epigenetic demethylation of the Treg-specific demethylation region (TSDR)^5^. In scurfy mice, loss-of-function mutations in *Foxp3* result in the development of a fatal autoimmune disorder^6^ while in humans *FOXP3* mutations lead to an X-linked disorder, IPEX (immunodysregulation polyendocrinopathy enteropathy X-linked)^7–9^. In humans, high CD25 cell surface expression in combination with low or absent CD127 expression is a useful marker for the distinction of Tregs from effector CD4^+^ T cells^10,11^.

Whether Tregs are a phenotypically and functionally stable population is under debate^12–14^. Elegant experiments using tracer mice containing FOXP3-GFP-Cre crossed with Rosa26-YFP have demonstrated instability in FOXP3 expression in a substantial percentage of Tregs when transferred into T cell-deficient mice^15,16^. However, others have demonstrated stable FOXP3 expression under homeostatic and autoimmune inflammatory conditions using similar tracer mice approach^17^, suggesting that the stability of the Treg linage may be regulated by environmental cues. For example, pro-inflammatory cytokines have been shown to cause Tregs to adopt a phenotype more characteristic of effector CD4^+^ T cells, downregulating FOXP3, losing their suppressive function and secreting pro-inflammatory cytokines^18,19^. Clinical examples of this plasticity are also evident, with IL-17-expressing Tregs increased in psoriasis, inflammatory bowel disease and rheumatoid arthritis, compared to healthy individuals^20–26^.

An important regulatory mechanism for Treg stability is co-stimulation, since it provides important signaling for Treg activation, survival, expansion, and acquisition of effector functions upon antigen recognition^27,28^. The CD27 co-stimulatory receptor is constitutively expressed on a small proportion of natural killer (NK) cells, memory B cells and resting CD8^+^ and CD4^+^ T cells, including CD4^+^FOXP3^+^ Tregs^29^. CD27 is expressed on thymocytes as early as the double positive stage of development^30^, and a role for CD27 in rescuing Tregs from apoptosis during clonal deletion in the thymic medulla has been reported^31^. In T cells, CD27 expression is increased on activation but then downregulated after prolonged stimulation^32^. CD27 expression is also lost on fully differentiated effector T cells, although central memory T cells retain CD27 expression^32,33^. Expression of CD70, the unique ligand for CD27, is tightly controlled and upregulated exclusively upon activation on T cells, B cells and certain subsets of dendritic cells (DCs)^34,35^. CD70 expression is very limited in the steady state, although increased levels of CD70 have been reported to be associated with inflammatory conditions such as chronic viral infection, cancer, or autoimmune disease^36–43^.

Ligation of CD27 on T cells with CD70 on antigen presenting cells (APCs) promotes T cell activation^44^, influences CD4^+^ T cell subset differentiation^31,45,46^ and is essential for the generation of antigen-specific T cell immunity by enhancing the survival of activated T cells^47–50^. Highlighting the importance of this interaction, genetic mutations in CD27 or CD70 in humans can result in persistent symptomatic EBV infection and EBV-associated lymphoproliferative disorders^51–56^. It has been previously shown that CD27 expression on human Tregs correlates closely with suppressive potency^57–60^. The mechanisms for this are unclear, although it has been proposed that CD27 on Tregs ligates CD70 on DCs, reducing the access of conventional T cells (Tconv) to this co-stimulatory molecule^61^. More recently, CD27 signaling has been shown to impair the conversion of tissue resident mouse Tregs into Th17 cells^22^. Moreover, several transcriptomic studies have demonstrated an increased expression of CD70 mRNA in human Tregs compared with conventional CD4^+^ T cells^62,63^.

CD27 signaling is sufficient to activate T cells when combined with T cell receptor (TCR) stimulation^47,49,64^. However, it is unclear how CD70 expressed on other cell types, such as Tregs, affects T cell function. In this study, we examine the role of the CD27/CD70 co-signaling axis in Treg function and how it is altered after prolonged Treg stimulation. We assess the relationship between CD27/CD70 and Treg fitness, showing that selection of CD27^+^CD70^−^ cells after Treg expansion enables the enrichment of potently suppressive cells with a transcriptome characteristic of Tregs. We also show that the prolonged expansion of human Tregs leads to stable gain of CD70 expression with associated loss of CD27, which in turn provides co-stimulatory signaling to CD27-expressing Tconv cells. Moreover, we show that the targeting of CD70 using monoclonal antibodies or genetic editing tools preserves the function of expanded Tregs, providing a method that may be incorporated into future cell therapy production protocols.

## Results

### CD27 and CD70 define distinct human Treg subpopulations

**Fig. 1:**
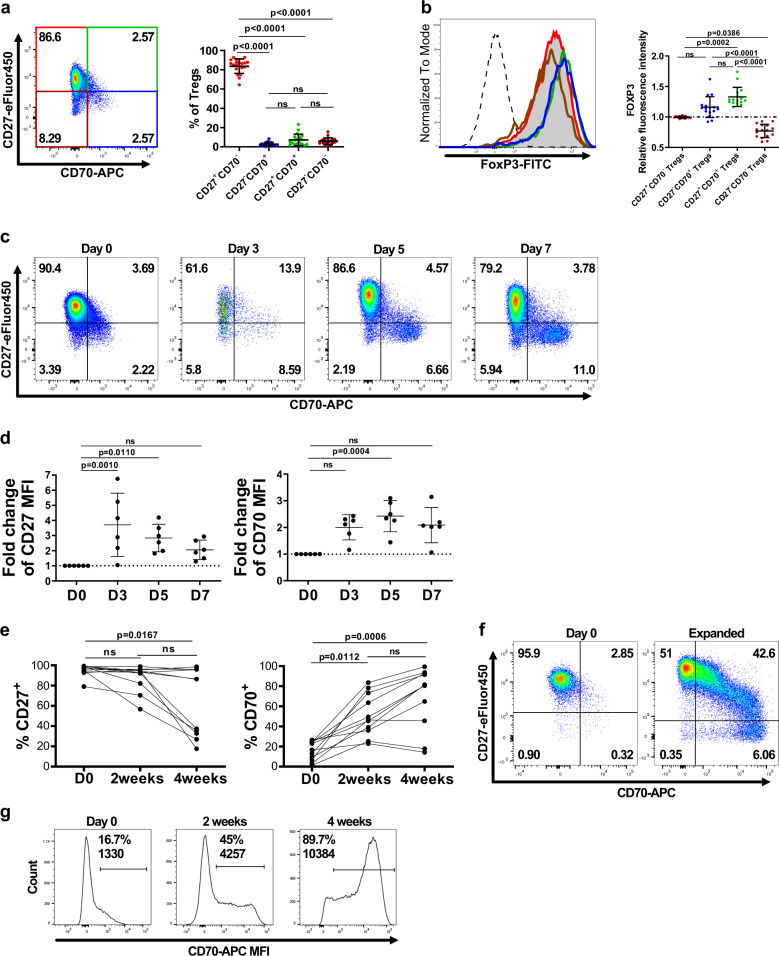
CD27 and CD70 define distinct human Treg subpopulations. **a** Expression of CD27 and CD70 was analyzed on freshly isolated CD4^+^CD25^+^CD127^−/low^ Tregs. Left panel: Representative FACS plots from one donor. Figures display percentages. Right panel: *n* = 19 independent donors. Data were analyzed using a Kruskal–Wallis test with Dunn’s post-test for multiple comparisons and are represented as mean ± SD. **b** FOXP3 expression was measured within the total Treg population or within different Treg subsets defined by CD27 and CD70 expression. Left panel: Representative FACS plots from one donor. Right panel: FOXP3 fluorescence intensity normalized to the total Treg population in different Treg subsets (*n* = 16 independent donors). Mean ± SD, Kruskal–Wallis test with Dunn’s post-test for multiple comparisons. **c**, **d**: **c** Dot plots for one representative donor in which expression of CD27 and CD70 was measured at different time points on Tregs stimulated with anti-CD3/anti-CD28 coated beads at a ratio of 1:5 beads to cells in the presence of rhIL-2 (250U/ml). Figures show percentages. **d** CD27 and CD70 fluorescence intensity was analyzed within the CD27^+^CD70^−^ or the CD27^−^CD70^+^ Treg populations, respectively, at different time points and normalized to levels prior stimulation. Data represented as mean ± SD. (*n* = 6 independent donors with 1–3 replicates per donor). Data were analyzed using a Friedman test with Dunn’s post-test for multiple comparisons. **e**–**g** Expression of CD27 and CD70 was analyzed on freshly isolated ex vivo Tregs or Tregs expanded for 2 or 4 weeks with anti-CD3/anti-CD28 coated beads at a ratio of 1:1 beads to cells in the presence of rhIL-2 (1000 U/ml). **e** Data show *n* = 11 independent donors analyzed using a Friedman test with Dunn’s post-test for multiple comparisons. **f** Representative plots for one donor. Figures show percentages. **g** Figures display percentage and fluorescence intensity of CD70^+^ cells for one representative donor. ns, non-significant.

We assessed expression levels of CD27 and CD70 on flow-sorted CD4^+^CD25^+^CD127^low/−^ human Tregs isolated at high purity levels from healthy donor PBMCs. The majority of these ex vivo Tregs were CD27^+^CD70^−^ (64.4–92.7%), with a variable, but minor, subpopulation expressing CD70 (Fig. 1a and Supplementary Data 1). Distinct CD27 and CD70 expression patterns were associated with different levels of FOXP3 expression (Fig. 1b and Supplementary Data 1). When normalized to the total Treg population, CD27^+^CD70^+^ and CD27^−^CD70^+^ subsets displayed a higher intensity of FOXP3 expression, while CD27^−^CD70^−^ and CD27^+^CD70^−^ subsets had a lower or similar level of FOXP3 expression, respectively (Fig. 1b and Supplementary Data 1).

Upon activation, there is dynamic expression of CD27 and CD70 in Tconv cells^32^. To assess whether this is also a feature in Tregs, we activated sorted CD4^+^CD25^+^CD127^low/−^ Tregs with anti-CD3/anti-CD28 coated beads and recombinant human IL-2 (rhIL-2) over a short period of time. Analysis of expression patterns revealed a reciprocal relationship between CD27 and CD70 on activated Tregs (Fig. 1c). Both CD27 and CD70 expression was increased after 3 or 5 days of stimulation (Fig. 1d and Supplementary Data 1). By 7 days, the frequency of CD70-expressing cells increased even further, forming a distinct subpopulation (Fig. 1c).

To investigate how prolonged stimulation may alter CD27 and CD70 expression, the phenotype of Tregs was examined following stimulation and expansion for 2–4 weeks. While initial levels of CD27 expression were high pre-expansion, after expansion there was a progressive reduction of expression (Fig. 1e, f and Supplementary Data 1). Conversely, CD70 expression levels were initially low and then increased after expansion (Fig. 1e–g and Supplementary Data 1). Following expansion, distinct Treg subpopulations could be identified by their pattern of CD27 and CD70 expression (Fig. 1f). Although transient CD70 expression upon activation has been previously described in other cell types^34,35^, we found that after long-term expansion Tregs maintained an unusually high and stable level of CD70 expression (Fig. 1g).

### Prolonged expansion reduces the suppressive capacity of Tregs

**Fig. 2:**
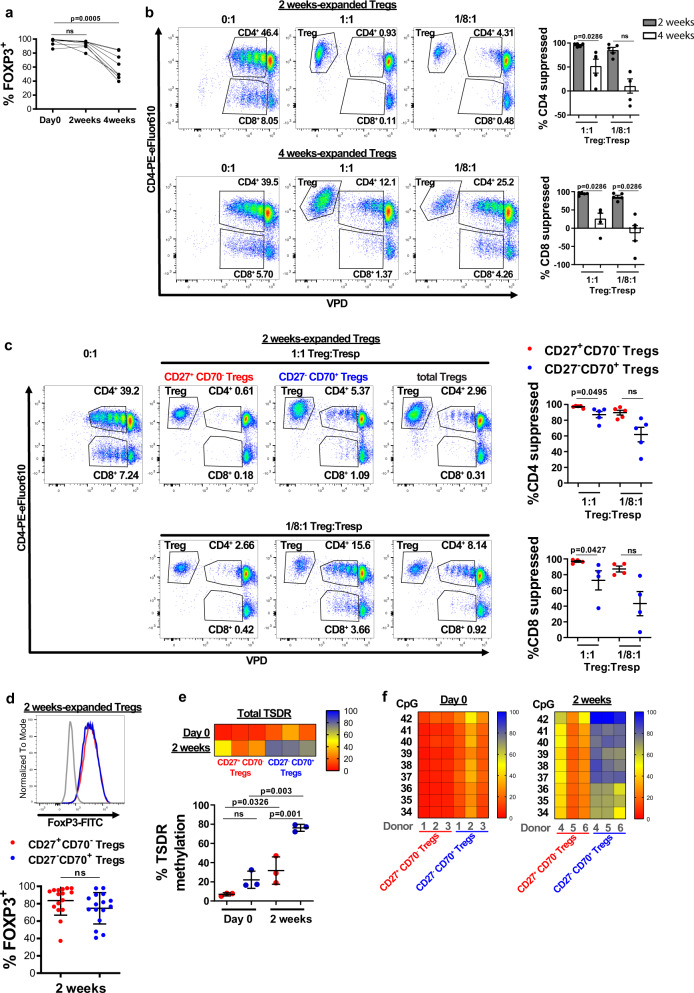
Prolonged expansion reduces the suppressive capacity of Tregs. CD4^+^CD25^+^CD127^−/low^Tregs were cultured in vitro with anti-CD3/anti-CD28 coated beads and rhIL-2 before analysis of **a** FOXP3 expression (*n* = 8 donors, Friedman test with Dunn’s post-test for multiple comparisons) and **b** in vitro Treg suppressive capacity, assessed as suppression of responder T cell (Tresp) proliferation. Left panel: percentage of VPD-stained CD4^+^ or CD8^+^ cells in the absence of Tregs (0:1) and in the presence of Tregs at a 1:1 or 1/8:1 Treg:Tresp ratios shown for one representative donor. Right panel: percentage suppression of CD4^+^ and CD8^+^Tresp calculated based on division index as described in “Methods” section. *n* = 5 donors (three replicate wells for each donor). Mean ± SEM, Friedman test and Dunn’s post-test for multiple comparisons. **c** In vitro suppressive capacity of CD27^+^CD70^−^ and CD27^−^CD70^+^ sorted Tregs. Left panel: Data from one representative donor. Right panel: Percentage suppression of CD4^+^ and CD8^+^ Tresp for 4–5 independent donors with each data point showing the mean of three replicate wells for each donor; calculated based on division index. Mean ± SEM, Kruskal–Wallis test with Dunn’s post-test for multiple comparisons. Red: CD27^+^CD70^−^Tregs; Blue: CD27^−^CD70^+^Tregs, both sorted after 2 weeks of expansion. **d** FOXP3 was analyzed in CD27/CD70 Treg subsets after 2 weeks of expansion. Top panel: histogram displays one representative donor (gray: isotype, red: CD27^+^CD70^−^ Tregs, blue: CD27^−^CD70^+^Tregs). Bottom panel: *n* = 16 independent donors. Mean ± SD, Mann–Whitney test. **e**, **f** DNA methylation pattern of the Treg-Specific Demethylation Region (TSDR) was assessed in CD27^+^CD70^−^ and CD27^−^CD70^+^Treg subsets from matched donors, sorted from freshly isolated and 2 weeks in vitro expanded CD4^+^CD25^+^CD127^−/low^Tregs. **e** Average methylation rate for 9 CpG motifs of the TSDR (*n* = 6 different donors). Top panel: Each box represents the percentage of methylation for each donor. Bottom panel: each dot denotes a donor. Mean ± SD, one-way ANOVA with Tukey post-test. **f** Each box represents the percentage of methylation of a single CpG residue for each donor at each time point (Left panel: day 0; right panel: 2 weeks) (*n* = 3 donors matched for each CD27/CD70 Treg subset). Bar colors designate: red: 0% methylation; yellow: 50% methylation; blue: 100% methylation. ns, non-significant.

We next assessed whether the changes in CD27 and CD70 expression were also associated with changes in Treg function. Pre-expansion, Tregs expressed high levels of FOXP3, and while this was largely maintained during the first 2 weeks of expansion, after 4 weeks there was a significant reduction in FOXP3 expression (Fig. 2a and Supplementary Data 1). Similarly, on assessment of Treg function, we found suppressive potency to be maintained after 2 weeks of expansion but reduced after 4 weeks (Fig. 2b and Supplementary Data 1).

We hypothesized that the loss in suppressive function was secondary to the expansion-related changes in CD27 and CD70 expression. On examination of the specific function of CD27^+^CD70^−^ or CD27^−^CD70^+^ Tregs after 2 weeks of expansion, we found the CD27^+^CD70^−^ Tregs to be significantly more potent at high but not low ratios of Tregs to responder cells (Fig. 2c and Supplementary Data 1). This was largely unrelated to any differences in FOXP3 expression (Fig. 2d and Supplementary Data 1). We then analyzed the methylation state of the TSDR region in sorted CD27^+^CD70^−^ and CD27^−^CD70^+^ Treg subsets from freshly isolated or 2-week expanded Tregs. In freshly isolated Tregs, both Treg subsets had a highly demethylated TSDR, confirming their high purity (Fig. 2e and Supplementary Data 1). Upon 2 weeks of expansion, the TSDR remained largely demethylated in CD27^+^CD70^−^ Tregs, whereas CD27^−^CD70^+^ Tregs developed a hypermethylated TSDR (Fig. 2e and Supplementary Data 1). Interestingly, the methylation pattern in expanded CD27^−^CD70^+^ Tregs was not equal across different CpG motifs (Fig. 2f and Supplementary Data 2).

Importantly, although CD4^+^CD25^+^CD127^−/low^ cells may include a small number of contaminating Tconv cells, these contaminants are unlikely to be the CD27^−^CD70^+^ cells. While activated effector T cells may transiently express FOXP3, we found significantly higher FOXP3 expression in CD27^−^CD70^+^ Tregs compared to CD27^−^CD70^+^ Tconv cells after 2 weeks of in vitro expansion (Supplementary Fig. 1), indicating that CD27^−^CD70^+^ Tregs are a separate population to effector T cells that upregulate FOXP3.

### Expanded CD27^+^CD70^−^ Tregs retain suppressive potency

**Fig. 3:**
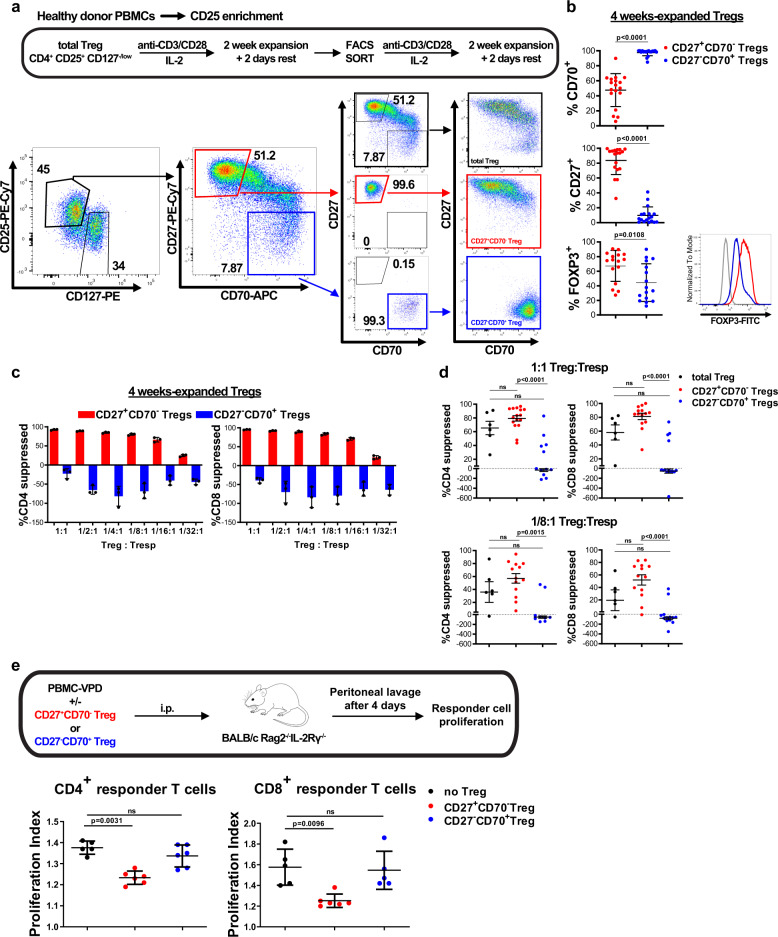
Expanded CD27^+^CD70^−^ Tregs retain suppressive potency. **a** Schematic overview of Treg expansion strategies. CD4^+^CD25^+^CD127^−/low^ Tregs were flow sorted from healthy donor PBMCs and expanded in vitro over two rounds of 7 days stimulated with anti-CD3/anti-CD28 coated beads (at a ratio of 1 bead to 1 cell) and rhIL-2 (1000U/ml) and rested for 2 days with a low concentration of rhIL-2 (250U/ml) and without stimulating beads. Tregs expanded for 2 weeks were flow sorted into CD27^+^CD70^−^ and CD27^−^CD70^+^ subsets. Total Tregs (black), flow sorted CD27^+^CD70^−^ Tregs (red) and flow sorted CD27^−^CD70^+^ Tregs (blue) were then cultured for a further 16 days before analysis. Dot plots from one representative donor are shown. Figures display percentages. **b** Left panel: FOXP3, CD27 and CD70 expression levels on expanded and then flow sorted CD27^+^CD70^−^ and CD27^−^CD70^+^ Tregs after a further 2 weeks of expansion. *n* = 16–18 independent donors, analyzed using a Mann–Whitney test. Data are represented as mean ± SD. Right panel: Histogram of FOXP3 expression levels for one representative donor (gray: isotype, red: CD27^+^CD70^-^ Tregs, blue: CD27^-^CD70^+^ Tregs). **c**, **d**, **c** In vitro suppression assay of 4 week-expanded Treg populations in one representative donor at multiple Treg:Tresp ratios. Data shown are mean ± SD. **d** In vitro suppression assays from 14 independent donors at 1:1 (top) and 1/8:1 (bottom) ratios of Treg to Tresp. Each data point represents the mean of three replicate wells from each donor. Mean ± SEM is depicted. Black: total Treg, red: CD27^+^CD70^−^, blue: CD27^−^CD70^+^ Tregs. Data were analyzed using Kruskal–Wallis test with Dunn’s post-test. **e** The in vivo suppressive capacity of CD27/CD70 Treg subsets was assessed by transplanting VPD-labeled PBMCs together with 4-week expanded CD27^+^CD70^−^ or CD27^−^CD70^+^ Tregs (5:1 PBMC:Treg ratio) into the peritoneal cavity of immunodeficient mice. Top: Schematic representation of the experimental plan. Bottom: The proliferation index of PBMCs was studied by flow cytometry. Data were analyzed using a Kruskal–Wallis test with Dunn’s post-test for multiple comparisons and are represented as mean ± SD. A representative experiment of 1 cell donor out of 2 (4–5 mice per group per experiment, each mouse is represented as a data point). ns, non-significant.

We next investigated whether re-sorting cells based on CD27 and CD70 expression at 2 weeks, followed by further expansion would address the loss of suppressive potency that develops between weeks 2 and 4 of expansion. Sorted Tregs were therefore expanded for 2 weeks and then flow sorted into CD27^+^CD70^−^ and CD27^−^CD70^+^ populations before continuing expansion for a further 2 weeks (Fig. 3a). While sorted CD27^−^CD70^+^ Tregs maintained their phenotype for the 2 subsequent weeks of expansion, CD27^+^CD70^−^ Tregs upregulated CD70 (Fig. 3b and Supplementary Data 1). Moreover, in approximately half of the analyzed donors, downregulation of CD27 was observed in re-stimulated CD27^+^CD70^−^ Tregs (Fig. 3b and Supplementary Data 1). FOXP3 expression was significantly higher in expanded CD27^+^CD70^−^ cells than CD27^−^CD70^+^ cells (Fig. 3b and Supplementary Data 1), which correlated with the TSDR demethylation at 2 weeks of expansion (Fig. 2e). Tregs with high CD27 expression tended to retain high levels of FOXP3 expression, while CD70 expression negatively correlated with FOXP3 expression levels (Supplementary Fig. 2).

On functional analysis, CD27^+^CD70^−^ Tregs were highly efficient at inhibiting CD4^+^ and CD8^+^ T cell proliferation and were consistently more potent than CD27^−^CD70^+^ expanded cells at all ratios tested (Fig. 3c, d and Supplementary Data 1). When comparing suppressive potency with CD27/CD70 expression in 4 week-in vitro expanded Tregs, we found no significant correlation between percentage of suppression of proliferation of CD4^+^ or CD8^+^ responder T cells and CD27 or CD70 expression, although a trend was observed (Supplementary Fig. 3).

Moreover, in the majority of donors analyzed, CD27^−^CD70^+^ Tregs actively promoted proliferation of CD4^+^ and CD8^+^ T cells (Fig. 3d and Supplementary Data 1). Although being consistently more potent than CD27^−^CD70^+^ Tregs, there was some heterogeneity in the suppressive activity of expanded CD27^+^CD70^−^ cells according to donor. We considered whether impaired suppression within the expanded CD27^+^CD70^−^ cells was due to the presence of cells that had downregulated CD27 and upregulated CD70. In support of this, we found that re-sorted CD27^high^CD70^−^ cells demonstrated significantly enhanced suppressive activity than the total population of CD27^+^CD70^−^ expanded cells (Supplementary Fig. 4).

To assess the increased potency of the CD27^+^CD70^−^ Treg subset in vivo, we performed a human leukocyte proliferation assay in immunodeficient mice. Violet proliferation dye (VPD)-labeled human PBMCs were transferred into mice together with human CD27^+^CD70^−^ or CD27^−^CD70^+^ Tregs which were expanded in vitro for 4 weeks. CD27^+^CD70^−^ Tregs, but not CD27^−^CD70^+^ Tregs, were able to suppress CD4^+^ and CD8^+^ T cell proliferation in vivo (Fig. 3e, Supplementary Fig. 5 and Supplementary Data 1).

### Transcriptome differences between CD27^+^CD70^−^ and CD27^−^CD70^+^ expanded Tregs

**Fig. 4:**
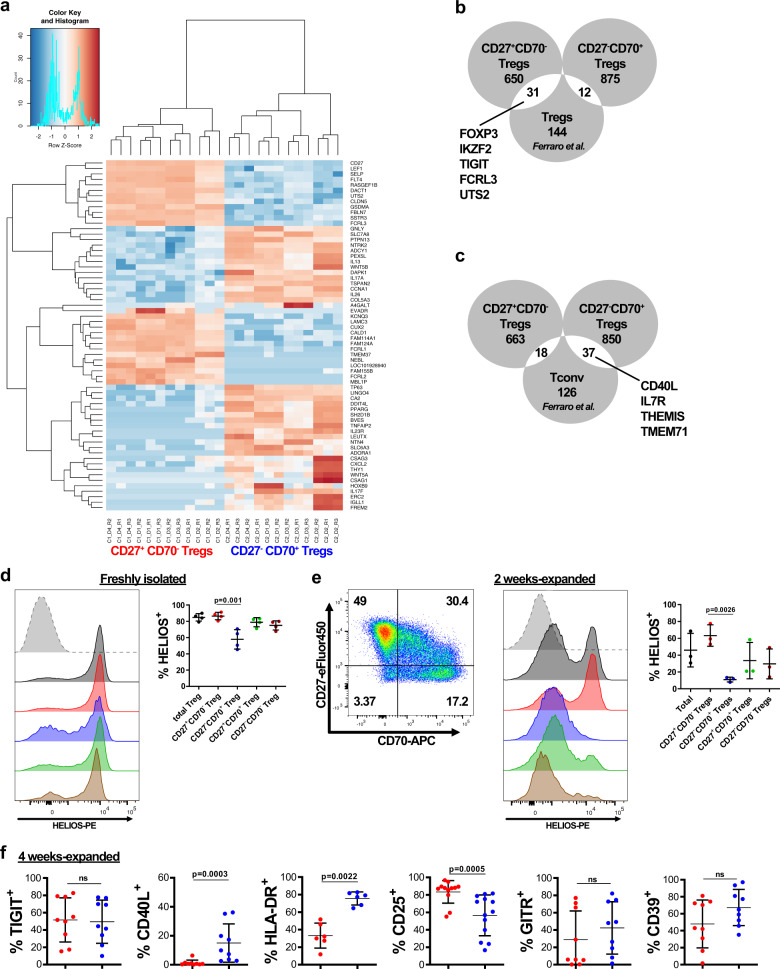
Transcriptome differences between CD27^+^CD70^−^ and CD27^−^CD70^+^ expanded Tregs. Thirty-two days-expanded Tregs (as shown in Fig. 3a) were re-sorted into CD27^+^CD70^−^ and CD27^−^CD70^+^ Tregs prior being assessed by RNA-seq analysis. **a** Heatmap and hierarchical clustering from RNA-seq data with Log2FC ≥ 6. The top 64 differentially expressed genes are shown. Data were analyzed using the edgeR package. **b** Venn diagram showing the overlap between genes overexpressed in Tregs^65^ and upregulated genes in CD27^+^CD70^−^ Tregs or CD27^-^CD70^+^ Tregs. **c** Venn diagram showing the overlap between genes overexpressed in Tconv^65^ and upregulated genes in CD27^+^CD70^-^ Tregs or CD27^−^CD70^+^ Tregs. **d**, **e**: **d** HELIOS expression was measured in freshly isolated total Tregs or within different Treg subsets defined by CD27 and CD70 expression. (Left panel) Representative FACS plots of one donor (gray: isotype, black: total Tregs, red: CD27^+^CD70^−^ Tregs, blue: CD27^−^CD70^+^ Tregs, green: CD27^+^CD70^+^ Tregs, brown: CD27^−^CD70^−^ Tregs). (Right panel) Percentage of HELIOS^+^ cells in different CD27/CD70 Treg subsets. **e** HELIOS expression was analyzed in 2 week-expanded total Tregs or within different Treg subsets defined by CD27 and CD70 expression. Left panel: CD27/CD70 gating strategy. Figures show percentages. Middle panel: Representative FACS plots of one donor (gray: isotype, black: total Tregs, red: CD27^+^CD70^-^ Tregs, blue: CD27^−^CD70^+^ Tregs, green: CD27^+^CD70^+^ Tregs, brown: CD27^−^CD70^−^ Tregs). Right panel: Percentage of HELIOS^+^ cells in different CD27/CD70 Treg subsets. Mean ± SD is represented. *n* = 3–4 independent donors. Kruskal–Wallis test with Dunn’s post-test for multiple comparisons was conducted. Only significant statistical differences are shown. **f** CD25, GITR, CD39, TIGIT, CD40L, and HLA-DR protein expression was analyzed in 4 weeks expanded CD27^+^CD70^−^ Tregs or CD27^-^CD70^+^ Tregs. *N* = 6–13 independent donors. Data were analyzed by Mann–Whitney test and represented as mean ± SD. ns, non-significant.

Having shown differences in the function of expanded CD27^+^CD70^−^ and CD27^−^CD70^+^ Tregs, we sought to compare gene signatures between these two Treg populations. CD27^+^CD70^−^ and CD27^−^CD70^+^ Tregs expanded over an extended period (as depicted in Fig. 3a) were re-sorted into CD27^+^CD70^−^ and CD27^−^CD70^+^ cells and analyzed by RNA sequencing (RNA-seq) to assess for differences in the transcriptomes (four independent blood donors). Global examination of the data by principal component analysis revealed the existence of two separate clusters of sample replicates (Supplementary Fig. 6a). In total, 1568 genes were differentially expressed between both Treg subsets (absolute Log2FC ≥ 2 and adjusted *p* value < 0.01); 681 transcripts were upregulated and 887 downregulated in CD27^+^CD70^−^ Tregs compared to CD27^−^CD70^+^ Tregs (Supplementary Data 3). Figure 4a contains the top 64 most differentially expressed genes (absolute Log2FC ≥ 6). Gene ontology (GO) term enrichment analysis of differentially expressed genes identified specific molecular pathways altered in both Treg subsets (Supplementary Fig. 6b). A large proportion of highly differentially expressed loci were related to cytokine and chemokine signaling, with signaling by interleukins, IL-4 and IL-13 signaling, IL-10 signaling, and chemokine receptor binding chemokine pathways being among the most enriched pathways. IL-17A, the effector cytokine for Th17 function, appeared as the most differentially expressed gene between CD27^+^CD70^−^ and CD27^−^CD70^+^ Tregs, being expressed ten times more in the CD27^−^CD70^+^ Treg subset (Fig. 4a and Supplementary Data 3). Overexpression of IL-17A was confirmed by measuring cytokine levels in cell culture supernatant and by intracellular cytokine staining (Supplementary Fig. 7).

We then compared the transcriptome profile of CD27/CD70 Treg subsets with a previously identified Treg-specific gene signature^65^. Ferraro et al. have identified 368 genes differentially expressed between Tregs and Tconv, with 187 and 181 genes upregulated in Tregs or Tconv, respectively^65^. From 187 genes specifically upregulated in Tregs, we found 31 genes that were overexpressed in CD27^+^CD70^−^ Tregs (and included *FOXP3, IKZF2, TIGIT, FCRL3, UTS2*) and 12 genes in CD27^−^CD70^+^ Tregs (Fig. 4b and Supplementary Data 4). Of the 181 genes upregulated in Tconv, 18 were overexpressed in CD27^+^CD70^−^ Tregs and 37 were overexpressed in CD27^−^CD70^+^ Tregs, including *CD40L, IL7R, THEMIS*, and *TMEM71* (Fig. 4c). Interestingly, other genes of the so called “Treg-specific gene signature”^65,66^, such as *CTLA-4, IL2RA, GITR* and *ENTPD1* (CD39) were not differentially expressed between CD27/CD70 Treg subsets. These results indicate that expanded CD27^+^CD70^−^ Tregs have a gene profile more closely related to the previously identified Treg gene signature than CD27^−^CD70^+^ Tregs, with some differences.

Canonical Treg-signature genes in the CD27^+^CD70^−^ cell population included typical markers associated with Treg biology such as *FOXP3, IKZF2* (HELIOS), and *TIGIT* (Fig. 4b). In freshly isolated Tregs, HELIOS protein expression was significantly lower in CD27^−^CD70^+^ than CD27^+^CD70^−^ Tregs (Fig. 4d and Supplementary Data 1). The same trend in HELIOS protein expression was observed in Tregs expanded for 2 weeks (Fig. 4e and Supplementary Data 1). Despite the fact that differences in TSDR demethylation and HELIOS expression were apparent after 2 weeks of expansion (Figs. 2e, f,  4e), FOXP3 expression and suppressive function were mostly unaltered between CD27^+^CD70^−^ Tregs and CD27^−^CD70^+^ Tregs at two weeks (Fig. 2c, d). Since it is only after 4 weeks of expansion that functional changes are apparent (Fig. 3e–d), we studied the expression of Treg-associated markers in these long-term expanded cells. Significant differences were found in CD25, CD40L, and HLA-DR expression, but not in GITR, CD39 or TIGIT, in the different CD27/CD70 Treg subsets (Fig. 4f and Supplementary Data 1).

### CD27^+^CD70^−^ Treg suppressive function does not rely on CD27 signaling

**Fig. 5:**
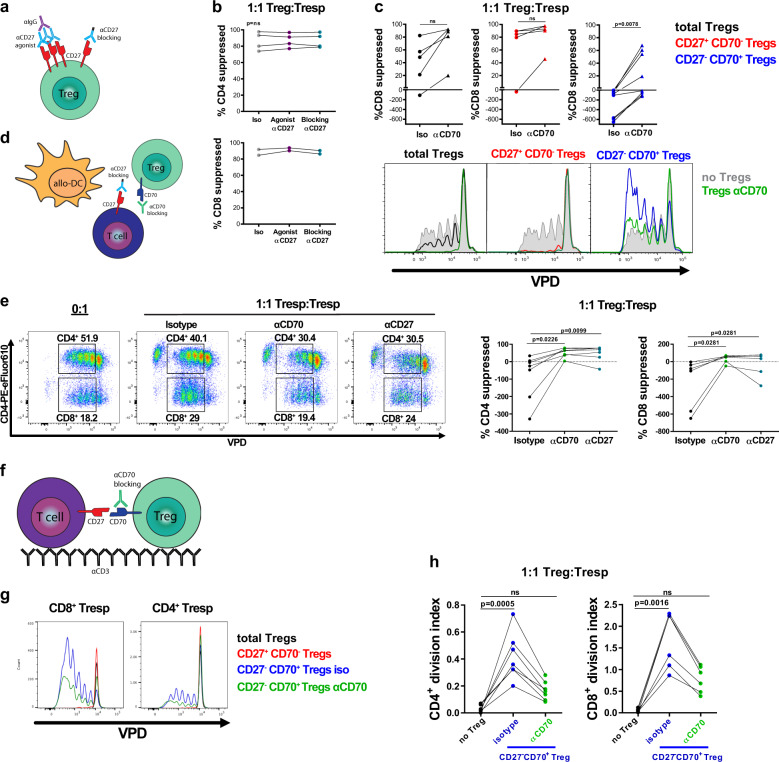
The CD27/CD70 co-stimulatory pathway contributes to Treg function. Data shown for 4 week-expanded Tregs (sorted as total Tregs, expanded for 2 weeks, sorted by CD27/CD70, then expanded for 2 weeks). **a**, **b**: **a** Cartoon depicting Treg CD27 targeting strategies. Tregs were pre-incubated for 1 h with Fc-mutated blocking anti-CD27 mAb or non-mutated anti-CD27 mAb, followed by 1 h cross-linking with anti-human Ig antibody, or isotype matched antibodies. **b** Percentage suppression of CD4^+^ and CD8^+^ T cell proliferation by Tregs pre-incubated with anti-CD27 mAbs in 4 donors. CD8^+^T cells did not proliferate enough to perform analysis in 2 out of 4 donors. Friedman test with Dunn’s post-test for multiple comparisons. **c** Top panel: percentage suppression of CD8^+^Tresp cell proliferation by Treg subsets pre-incubated with anti-CD70 mAb or isotype antibody. *n* = 5–8 independent cell donors, Wilcoxon matched-pairs test. Bottom panel: proliferation of VPD-labeled CD8^+^Tresp co-cultured with Tregs pre-incubated with isotype or blocking anti-CD70 mAb, one representative donor. **d**, **e**: **d** Cartoon depicting CD27/CD70 blocking strategies. Tregs were pre-incubated with anti-CD70 blocking mAb or matched isotype Ab. Alternatively, soluble Fc-mutated blocking anti-CD27 mAb was added to the suppression assay to block CD27 on Tresp. **e** Left panel: Percentage of dividing VPD-stained CD4^+^ or CD8^+^ cells in the absence of Tregs (0:1) and in the presence of CD27^-^CD70^+^ Tregs pre-incubated with isotype or anti-CD70 blocking mAb (1:1 Treg:Tresp). Right panel: Percentage suppression of proliferation of CD4^+^ and CD8^+^ Tresp. *n* = 7 independent donors, Friedman test with Dunn’s post-test. **b**, **c**, **e** Graphs calculated based on division index and formula described in “Methods” section. **f**–**h**: **f** VPD-labeled Tresp were co-cultured with CD27^+^CD70^−^Tregs or CD27^−^CD70^+^Tregs pre-incubated with isotype or anti-CD70 mAb in wells containing plate-bound anti-CD3 mAb (1 µg/ml). **g** Proliferation of CD8^+^ or CD4^+^Tresp in the absence of Tregs or when co-cultured with Treg subsets for one representative donor out of 5. **h** Division index of proliferating CD4^+^ and CD8^+^Tresp co-cultured with CD27^−^CD70^+^Tregs pre-incubated with isotype mAb or blocking anti-CD70 mAb. *n* = 7 donors, Friedman test with Dunn’s post-test. **b**, **c**, **e**, **h** Each data point represents the mean of three replicate wells for each independent cell donor. ns, non-significant.

We next explored the role that CD27 plays in the suppressive activity of Tregs. For these studies, we used anti-CD27 monoclonal antibodies (mAbs) where the precise function depends on the presence or absence of cross-linking, which can be provided by Fc receptors or artificially^64,67^. Using these antibodies cross-linked with goat anti-human IgG results in agonistic function, whereas an Fc-mutated anti-CD27 mAb in which cross-linking by Fc receptors is prevented results in antagonistic activity^67^ (Fig. 5a).

To investigate the role of CD27 in the suppressive activity of Tregs, CD27^+^CD70^−^ Tregs were pre-incubated with anti-CD27 mAb provided under either activating or blocking conditions and assessed for their capacity to suppress T cell proliferation in vitro. Tregs pre-incubated in medium alone, isotype control, agonistic anti-CD27 mAb, or blocking anti-CD27 mAb showed similar suppressive potency (Fig. 5b and Supplementary Data 1), indicating that stimulation or blocking of CD27 signaling on Tregs does not have a significant effect on Treg suppressive activity in vitro.

### CD70 on Tregs provides co-stimulation to conventional T cells

In order to further explore the differences in suppressive potency between CD27^+^CD70^−^ and CD27^−^CD70^+^ Tregs, we investigated whether this was a result of CD70-mediated co-stimulation. Total unsorted, CD27^+^CD70^−^, or CD27^−^CD70^+^ Tregs were pre-incubated with anti-CD70 blocking mAb or matched isotype control antibody and their suppression was assessed in vitro. The effective binding of anti-CD70 blocking mAb was confirmed by FACS using an anti-CD70-fluorochrome labeled mAb that is sterically hindered by anti-CD70 blocking mAb (Supplementary Fig. 8). Blocking CD70 inhibited the pro-stimulatory effect of CD27^−^CD70^+^ Tregs and enhanced suppressive potency of total unsorted Tregs without affecting the activity of CD27^+^CD70^−^ Tregs (Fig. 5c and Supplementary Data 1). In some donors, CD27^−^CD70^+^ Tregs were only ever suppressive when CD70 was blocked (Fig. 5c).

To further demonstrate that CD70^+^ Tregs provide co-stimulatory signaling by binding CD27 on T cells, responder T cells were co-cultured with total Tregs or CD27^−^CD70^+^ Tregs and allogenic monocyte derived DCs (allo mo-DCs) in the presence of anti-CD27 blocking mAb (Fig. 5d). Blocking CD27 on responder T cells resulted in suppression of proliferation to a similar extent as blocking CD70 on Tregs (Fig. 5e and Supplementary Data 1).

To eliminate the possibility that anti-CD70 mAb dissociates from Tregs and subsequently inhibits T cell proliferation by binding to responder T cells or mo-DCs, we added anti-CD70 blocking mAb directly to the co-culture. In the absence of Tregs, proliferation of responder T cells was not impaired by CD70 blockade (Supplementary Fig. 9). When CD70^+^ Tregs were added to the cell co-culture that contained anti-CD70 blocking mAb, the pro-proliferative activity of the Tregs was neutralized (Supplementary Fig. 9). This suggests that the anti-CD70 antibody acts directly on Tregs and not on responder T cells or mo-DCs.

We next assessed whether CD70 expressed on Tregs provides sufficient co-stimulation to promote T cell proliferation in the absence of additional co-stimulatory help. Treg activity was therefore assessed in the absence of DC-driven stimulation. VPD-labeled responder T cells were co-cultured with CD27^+^CD70^−^ Tregs or CD27^−^CD70^+^ Tregs in wells containing plate-bound anti-CD3 mAb (Fig. 5f). Here, only CD27^−^CD70^+^ Tregs induced T cell proliferation, with CD8^+^ T cells appearing more responsive to CD27^−^CD70^+^ Treg stimulation in comparison to CD4^+^ T cells (Fig. 5g, h, Supplementary Fig. 10 and Supplementary Data 1). Similar to the effect observed by CD27^-^CD70^+^ Tregs, stimulation using agonist anti-CD27 mAb also increased T cell proliferation, especially within the CD8^+^ T cell compartment (Supplementary Fig. 11a). Moreover, CD27 stimulation increased expression of the anti-apoptotic Bcl-XL molecule and reduced the frequency of cell death in CD4^+^ and CD8^+^ T cells (Supplementary Fig. 11b, c).

We next explored the specificity of this response by pre-incubation of CD27^−^CD70^+^ Tregs with blocking anti-CD70 mAb prior to the subsequent culture with responder T cells. Although some proliferation of responder T cells still took place, blocking the CD27–CD70 interaction impaired the proliferative effect induced by CD27^−^CD70^+^ Tregs. Interestingly, the effect of blocking CD70 was not complete, suggesting the presence of additional pro-inflammatory mechanisms (Fig. 5h and Supplementary Data 1).

### CD70 knockout Tregs have enhanced suppressive potency

**Fig. 6:**
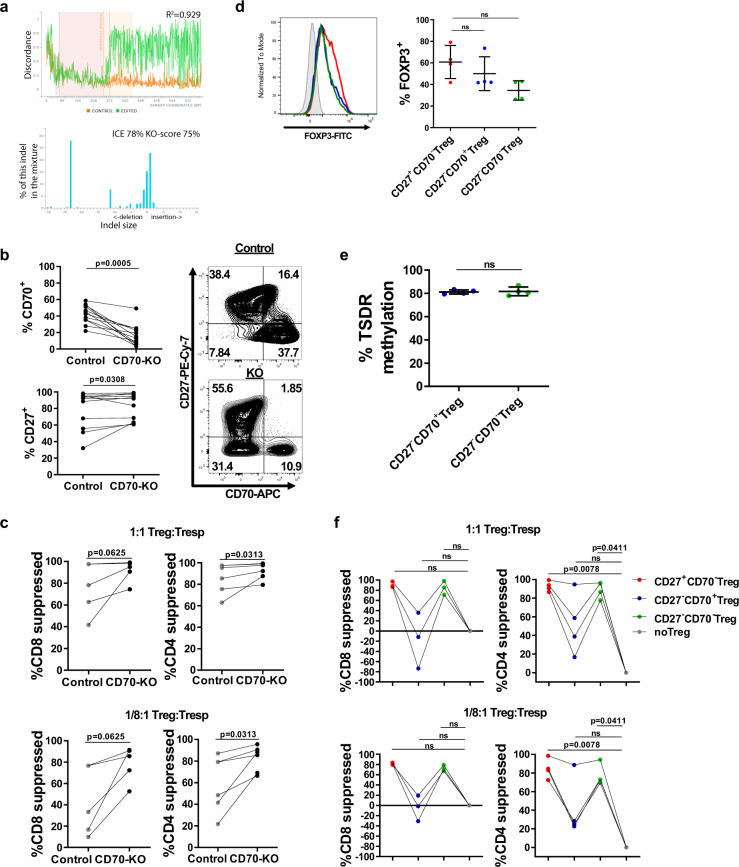
CD70 knockout Tregs display enhanced suppressive potency. **a** Genetic editing efficiency across the CD70 locus was confirmed by analysis using ICE algorithm. Top panel: Level of discordance between control (orange) and edited (green) genetic sequences. Sequences between control and edited samples match before the PAM sequence (denoted by a dotted line) and are discordant after the cut site. Bottom panel: Graph shows the distribution of the detected indels in the edited sample. *X*-axis depict the size of the indel and *Y*-axis shows the frequency of each deletion in the cell product. 1 sgRNA for a representative donor is shown. **b** Percentage of CD70 and CD27 expression in control and CD70-KO cell products. Assays were repeated in 12 independent experiments. Data were analyzed using a Wilcoxon matched-pairs test. **c** Treg suppressive capacity was assessed in vitro using autologous VPD-labeled CD3^+^CD25^−^ T cells as responders (Tresp) and allogenic mo-DCs as stimulators. Suppressive activity of control (non-edited) and CD70-KO cell products is represented for six independent experiments. Percentage suppression of proliferation of CD4^+^ Tresp and CD8^+^ Tresp is shown as mean ± SEM. Statistical significance was calculated using a Wilcoxon matched-pairs test. **d**–**f** CD70-KO Treg cell products were sorted by magnetic isolation or flow cytometry in CD27^+^CD70^−^, CD27^−^CD70^+^ and CD27^−^CD70^+^ cells subsets before assessing **d** FOXP3 expression, **e** DNA methylation pattern of the TSDR, and **f** suppressive activity. Data shown for 4 independent experiments. **d** Left panel: histogram displays one representative donor (gray: isotype, red: CD27^+^CD70^−^ Tregs, blue: CD27^−^CD70^+^ Tregs, green: CD27^−^CD70^−^ Tregs). Right panel: Statistical significance was calculated using a Friedman test with Dunn’s post-test for multiple comparisons. **e** Average methylation rate for nine CpG motifs of the TSDR. Error bars indicate mean ± SD. Statistical significance was determined by a Wilcoxon matched-pairs test. **f** Treg suppressive capacity was assessed in vitro. Percentage suppression of proliferation of CD4^+^ Tresp and CD8^+^ Tresp is represented. Statistical analyses performed using Friedman test with Dunn’s post-test for multiple comparisons. ns, non-significant.

To prove the detrimental role of CD70 in Treg suppressive activity, we next produced CD70-deficient human Tregs by disrupting CD70 expression using CRISPR (clustered regularly interspaced short palindromic repeats)-associated nuclease Cas9 technology. Molecular signatures of gene editing at the *CD70* locus were confirmed by PCR sequencing and ICE analysis in the total cell product (Fig. 6a and Supplementary Fig. 12). Most of the mutations comprised an insertion of 1 bp (22.9%) or deletion of 23 bp (21.46%), creating a knock out (KO)-score of 75% when analyzing the percentage of indels>20 bp or those resulting in frameshifts (Fig. 6a; bottom panel). Gene-edited Tregs were cultured in vitro for 2 weeks with anti-CD3 and anti-CD28 stimulation and in the presence of rhIL-2. Following this, we observed a significant reduction of CD70 and an increase of CD27 expression, together with the development of a CD27^−^CD70^−^ population in the gene edited group (CD70-KO) (Fig. 6b and Supplementary Data 1).

On functional assessment, CD70-KO Tregs had enhanced suppressive potency compared to control Tregs in controlling CD4^+^ T cell proliferation (Fig. 6c and Supplementary Data 1). Since the KO effect was not highly effective in all donors tested, we were able to sort different Treg subsets based on CD27 and CD70 expression from the total CD70-KO cell product (see sorting gate Supplementary Fig. 13). We analyzed each subset independently for FOXP3 expression, TSDR methylation and capacity to suppress T cell proliferation in vitro. No significant differences were observed in FOXP3 expression (Fig. 6d and Supplementary Data 1) or levels of TSDR methylation (Fig. 6e and Supplementary Data 1) due to CD70 silencing, indicating that CD70 does not control stability of FOXP3 expression. As previously shown, CD27^−^CD70^+^ Tregs displayed impaired suppression compared to CD27^+^CD70^−^ Tregs (Fig. 6f and Supplementary Data 1). Importantly, suppressive capacity was restored in the CD27^−^CD70^−^ Treg subset (which contained the CD70-KO Tregs) (Fig. 6f).

### CD70^+^ Tregs are present within resting CD4^+^ T cell populations in the peripheral blood

**Fig. 7:**
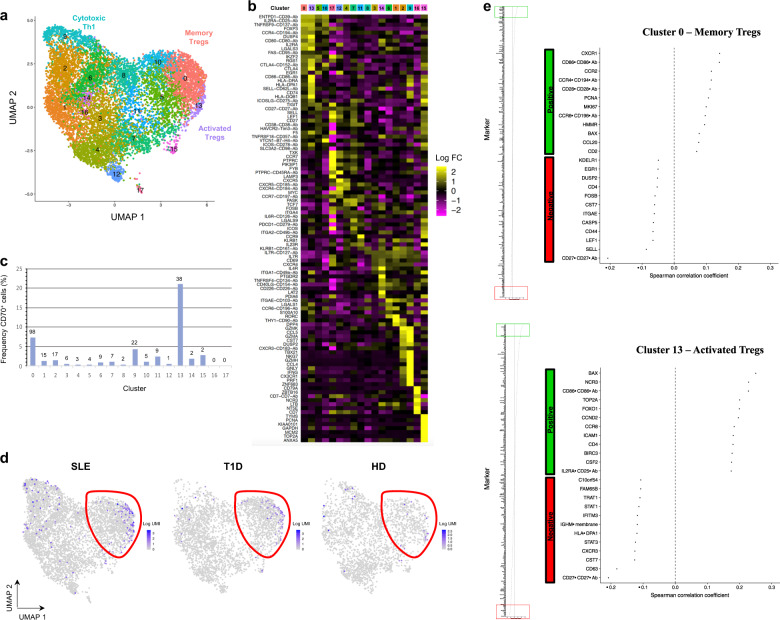
Identification of human CD70^+^ Tregs within the peripheral blood by integrated single-cell targeted simultaneous protein and mRNA analysis. Data from primary resting CD4^+^ cells from the peripheral blood from one systemic lupus erythematosus (SLE) patient, one type I diabetes patient (T1D) and one healthy donor (HD) and analysed using targeted scRNA-seq and protein quantification with BD Rhapsody technology from Trzupek et al.^68^ were re-analysed with focus on CD70 expression. A detailed description of this methodology and analysis of these cells can be found in the ref. ^68^**a** Unsupervised hierarchical clustering combining mRNA and protein expression data visualized as a Uniform Manifold Approximation and Projection (UMAP) plot depicting the clustering of memory/effector CD4^+^ T cells from all three donors integrated from two independent experiments. **b** Heatmap for each CD4^+^ cluster showing the top differentially expressed genes and proteins. **c** Analysis of CD70^+^ cell frequency within each UMAP cluster in **a**. The number of cells with ≥1 copy of CD70 is displayed for each cluster. The majority of CD70 mRNA-expressing cells fall within Cluster 0 (Memory Tregs) and Cluster 13 (Activated Tregs). **d** UMAP plots depicting relative expression of CD70 mRNA for each patient, with Treg clusters highlighted. **e** Correlation analysis (Spearman correlation coefficient) of CD70 and each assessed mRNA and protein target in the Treg Cluster 0 (top) and Cluster 13 (bottom), with an enhanced view of the top 12 most positively (green) or negatively (red) correlated genes.

We next aimed to map CD70^+^ Tregs using single-cell analysis in order to determine their respective representation within resting primary CD4^+^ T cells in humans. For accurate phenotyping of this heterogeneous population, we used a targeted multiomics single-cell RNA sequencing (scRNAseq) system in which both gene and protein expression are simultaneously quantified, as previously described^68^. We reanalyzed data presented in Trzupek et al.^68^ with focus on CD70 expression. Briefly, CD4^+^ T cells from the peripheral blood of one patient with systemic lupus erythematosus (SLE; *n* = 9708 cells), one patient with type 1 diabetes (T1D; *n* = 7042 cells) and one healthy donor (HD; *n* = 7197 cells) were studied. Unsupervised hierarchical clustering was performed in the combined mRNA and protein expression data, yielding distinct clustering of cells across both naive/memory and effector/Treg spectra^68^. Analysis of CD70 expression in this dataset demonstrated restricted expression in memory and effector populations (Supplementary Fig. 14), and therefore naive cells were excluded and a novel unsupervised hierarchical clustering was performed (Fig. 7a). This approach provided detailed functional compartmentalization of the heterogeneous memory CD4^+^ T cell population, which is evident by the differential of genes across each numbered cluster, with clusters 0, 13, 5, and 10 expressing Treg-signature genes, including *FOXP3* and *IKZF2* (HELIOS; Fig. 7b). Among all clusters, CD70 expression was highest within clusters 13, 0, and 9, which displayed transcriptional profiles of activated Tregs, memory Tregs and putative cytotoxic Th1 cells, respectively (Fig. 7c and Supplementary Fig. 15a). This presence of CD70^+^ cells within Treg clusters 0 and 13 was consistent across patients (Fig. 7d), and although a higher frequency was observed within the SLE patient, this was associated with a general increased frequency of Tregs and therefore not a specific enrichment. On analysis of genes correlating with CD70 expression within clusters 0 and 13, we found a differential overexpression of genes related to cell cycle progression (MKI67, PCNA, HMMR, and TOP2A), suggesting that CD70^+^ Tregs are actively proliferating (Fig. 7e). CD70 also correlated with CCR6 expression, a marker of IL-17-producing T cells (Supplementary Fig. 15b)^69^. Importantly, a clear negative correlation was observed with the relative expression of CD27, which was found to be the most negatively differentially regulated gene in clusters 0 and 13 (Fig. 7e and Supplementary Fig. 15b). Together, these data indicated that CD70^+^ Tregs are present within resting CD4^+^ T cells in the peripheral blood and express a gene and protein signature consistent with our in vitro observations.

## Discussion

In this study, we show that the prolonged stimulation of Tregs results in the development of a population of cells with a divergent gene signature and pro-inflammatory activity. These cells may be characterized principally by their expression of cell surface CD70. Prolonged stimulation is a feature of current Treg cell therapy production techniques and may also occur in pathological conditions in vivo^70,71^. Dysfunctional Tregs are strongly associated with clinical pathology^20–24,26^, and we therefore urge caution in the therapeutic infusion of Treg cell therapies intended to be immune suppressive if these products contain a significant proportion of CD70^+^ Tregs.

We have previously reported that expanded naive Tregs retain higher CD27 expression than memory Tregs^72^, and CD27 has been shown to positively correlate with suppressive potency^57,58,60^. Moreover, the immunosuppressive drug rapamycin, which is currently used for *ex vivo* expansion of clinical grade Tregs^73^, has been shown to preserve CD27 expression during in vitro Treg expansion^58^. Here, we demonstrate that freshly isolated Tregs have high CD27 and low CD70 expression and that both markers are upregulated following activation. However, upon prolonged stimulation in a significant portion of Tregs, CD27 is downregulated while CD70 expression is upregulated as a stably expressed marker, resulting in two distinct subpopulations: CD27^+^CD70^−^ and CD27^−^CD70^+^. Previous studies on *ex vivo* expanded human Tregs have demonstrated that Tregs maintain a regulatory phenotype and high suppressive potency after one or two cycles of expansion^74^. However, Hoffmann et al. showed that CD4^+^CD25^+^CD127^low/−^ Tregs display increased methylation within the TSDR region and decreased levels of FOXP3 expression after repetitive TCR stimulation^75^. In agreement with these data, our work shows that prolonged in vitro stimulation and expansion of flow-sorted highly pure CD4^+^CD25^+^CD127^low/−^ Tregs results in a final cell product that contains cells with unstable regulatory activity. Within this population, CD27^+^CD70^−^ Tregs share important elements of the transcriptome with the previously identified Treg-signature transcriptome^65^, and retain potent suppressive activity and a demethylated TSDR upon long-term in vitro stimulation. By contrast, CD27^−^CD70^+^ Tregs have a significantly impaired suppressive capacity after prolonged in vitro stimulation and, conversely, the ability to provide co-stimulatory signals to induce T cell proliferation. While our results suggest that FOXP3 regulates CD70 expression, published data comparing Tregs and Tconv cells^65,76,77^ or FOXP3 transduction in CD4^+^ T cells^78^ suggest no direct interaction between FOXP3 expression and CD70. We therefore propose that CD70 has the active role in regulating Treg suppressive activity by providing direct co-stimulatory signals to responder T cells.

It has been suggested that CD27^+^ Tregs may compete for CD70 binding on DCs, resulting in endocytosis and degradation of the CD27/CD70 complex^61^. Consequently, DCs are unable to provide CD70-mediated co-stimulation to responder T cells^61^. However, our experiments in which we blocked CD27 on Tregs indicate that CD27 does not play a non-redundant role in their suppressive activity. These findings are consistent with studies where Tregs from wild type and CD27^−/−^ mice were shown to be equally suppressive^31^. Moreover, Remedios et al. have shown that CD27 signaling limits the conversion of skin-resident Tregs into Th17 cells under an inflammatory environment driven by *Candida albicans* infection^22^. Indeed, in our experiments we have also shown that in humans IL-17 production is limited to the CD27^−^CD70^+^ Treg population. Interestingly, Remedios et al. found that while CD27 ligation prevented expression of Th17-associated molecules in Tregs, it did not affect FOXP3 or CD25 expression levels, suggesting that CD27 signaling does not increase Treg stability^22^. Here, we have shown that CD27^−^CD70^−^ Tregs (isolated from CD70-KO Treg cell products after in vitro expansion) are as potent as CD27^+^CD70^−^ Tregs at inhibiting T cell proliferation in vitro, further indicating that suppressive activity can be preserved in Tregs that are expanded over a prolonged period independently of CD27 expression.

Previous studies investigating CD70 co-stimulation provided by effector T cells have reported different outcomes under transient or constitutive antigen exposure in vivo. For example, in the presence of chronic viral infection, CD70 co-stimulation provides survival signals to antigen-activated T cells for their maintenance that ultimately leads to T cell exhaustion and defective memory immune responses^79,80^. However, during transient infections, CD70-driven co-stimulation enhances T cell immune responses without resulting in T cell exhaustion^79,80^. In line with these data, O’Neill et al. have described a role for T cell-derived CD70 co-stimulation restraining inflammatory T cell responses by inducing T cell apoptosis in mouse models of autoimmune inflammatory bowel disease (IBD) and GvHD^81^. Here, we have shown that CD70 is expressed by Tregs following activation and that CD70^+^ Tregs (stably expressing CD70) after prolonged in vitro stimulation, can provide sufficient co-stimulation to induce T cell activation and proliferation. In resting primary CD4^+^ T cells from the peripheral blood, CD70^+^ Tregs appear to reside within activated and memory Treg subpopulations where their gene expression signature is consistent with proliferating cells that downregulate expression of CD27. However, it is possible that CD70^+^ Tregs may promote different outcomes in vivo depending on the nature and precise context of the immune response. Due to the therapeutic potential of Treg cell therapy in transplantation and autoimmunity, both disorders in which there is persistent antigen presence, future work is needed to determine the role of CD70^+^ Tregs in vivo in both steady state and inflammatory states.

Interestingly, CD70^+^ Tregs primarily promoted proliferation of CD8^+^ T cells. CD27 co-stimulation has been identified as a key CD4^+^ T cell-delivered help signal to CD8^+^ T cells^82–84^. Here, antigen presenting-DCs upregulate CD70 upon interaction with CD4^+^ T cells via CD40L–CD40 interaction and transmit the help signal to trigger CD8^+^CD27^+^ T cell activation^82^. In our study, we show that Tregs induce CD4^+^ and CD8^+^ T cell proliferation via a CD27–CD70 interaction in the absence of DCs, indicating that CD4^+^CD70^+^ Tregs can provide direct co-stimulation to CD4^+^ and CD8^+^ T cells.

Genetic deletion or blockade of CD70 on Tregs or CD27 on T cells significantly enhanced Treg suppression, partly abolishing the pro-stimulatory effect of CD70^+^ Tregs. While contamination with effector T cells within CD70^+^ Tregs may be possible, CD27^−^CD70^+^ cells were already present within freshly isolated Tregs, had similar levels of TSDR demethylation as CD27^+^CD70^−^ Tregs, and expressed high FOXP3 levels. Moreover, CD27^−^CD70^+^ Tregs suppressed T cell proliferation after 2 weeks of expansion but lost this ability after 4 weeks. In addition, within the highly pure isolated CD27^+^CD70^−^ Tregs, some cells converted to CD27^−^CD70^+^ cells and the re-sorted CD27^high^CD70^−^ Tregs showed increased suppressive ability. The ability to convert some CD27^−^CD70^+^ into actively suppressive cells through the simple blockade of CD70 with an antibody argues against the possibility that these cells are contaminating effector cells. The last piece of evidence that supports Treg instability comes from our genetic editing experiments disrupting CD70 expression. Here, CD27^−^CD70^−^ Tregs isolated from a CD70-KO product were as suppressive as CD27^+^CD70^−^ Tregs, proving that CD70^+^ Tregs can exert potent suppressive capacity when CD70 function is disabled. These results further support our hypothesis of CD70 being a crucial regulator of Treg activity, although it is possible that other molecules identified by our RNA sequencing as differentially expressed could also play a role.

Monoclonal antibodies that target co-stimulatory and co-inhibitory molecules have a wide-range of established roles in the treatment of immune-mediated pathologies^85^. For cancer, the most promising are those that inhibit the checkpoint receptors PD-1 and CTLA-4, leading to increased antitumor T cell activity^86^. Conversely, CD28 antagonists designed as CTLA-4-Ig fusion proteins are effective at ameliorating symptoms in autoimmune disorders and at preventing transplant rejection^87^. The modulation of members of the tumor necrosis factor receptor family (TNFR) including CD40, OX40, and 4-1BB is also currently a focus of investigation^64,88–90^, with amplification of CD27 signaling being explored for cancer immunotherapy^67,91^. Our study provides a new understanding of human Treg stability and suggests that the CD27/CD70 axis could be exploited for regulating inflammatory and regulatory T cell responses. This could be approached through the direct treatment of patients with modulators of this pathway or manipulation of Tregs ex vivo for subsequent infusion. For example, the pro-proliferative effects of CD70^+^ Tregs can be prevented by monoclonal antibodies that block CD70 on Tregs or CD27 on T effector cells. Additionally, CD70-genetic ablation or the selection of ex vivo Tregs based on the CD27^+^CD70^−^ phenotype after expansion eliminates a major component of unstable, pro-stimulatory cells in the final product. We propose that depletion of CD70^+^ Tregs following in vitro expansion may be incorporated as an additional selection step into current clinical grade  production methods. Moreover, with the recent introduction of genetic editing tools into clinical trials, it is possible that deletion of CD70 on Tregs may be incorporated into GMP-compliant Treg expansion protocols.

## Methods

### Treg flow-sorting strategies and cell culture

Peripheral blood mononuclear cells (PBMCs) were isolated from blood cones obtained from healthy donors (NHS Blood and Transplant [NHSBT] UK) by LSM1077 (PAA Laboratories, Pasching, Austria) gradient centrifugation and then incubated with CD25^+^ Microbeads to derive CD25^+^ enriched cells using LS columns (Miltenyi Biotec, Bergisch Gladbach, Germany). CD4^+^CD25^+^CD127^−/low^ Tregs were isolated from CD25^+^ enriched PBMCs by FACS using a BD FACSAria I or II cell sorter.

Cells were cultured in complete media, composed of RPMI 1640 media supplemented with l-Glutamine, penicillin/streptomycin and sodium pyruvate (Sigma-Aldrich, St. Louis, MO, USA). For Treg expansion, 10% of human antibody pooled serum (Seralab, Haywards Heath, UK) (heat-inactivated for 20 min at 56 °C) was added to the medium. Tregs were expanded as described previously^92^. For in vitro assays, 10% of fetal calf serum (FCS, heat inactivated for 20 min at 56 °C) (Gibo, UK) was used instead of human serum. Cells were incubated at 37 °C, 5% carbon dioxide and >80% humidity. Expanded Tregs were stained with anti-CD27 PeCy7 (M-T271) and anti-CD70 APC (113-16) (BioLegend) antibodies in order to separate into CD27^+^CD70^−^ and CD27^−^CD70^+^ cells (Fig. 3a). Cell sorting was performed using a BD FACSAria I or II cell sorter.

### In vitro stimulation of Tregs

Sorted Tregs were activated with Dynabead Human T-activator anti-CD3/anti-CD28 beads (Thermo Fisher Scientific, MA, USA) in a 5:1 cell:bead ratio in complete media, in the presence of recombinant human IL-2 (rhIL-2) (250U/ml) (Novartis, Surrey, UK). For prolonged in vitro culture (16–36 days), Tregs were stimulated with anti-CD3/anti-CD28 beads every 7 days in a 1:1 cell:bead ratio in complete media in the presence of rhIL-2 (1000U/ml). Cells were split, and media changed as required. Tregs were rested without beads and with 250 U/ml rhIL-2 for 2 days every 14 days of culture.

### Flow cytometric phenotyping

Cells were stained with 7-AAD viability dye, anti-CD4 PE-eFluor 610 (RPA-T4), anti-CD27 eFluor 450 (O323), anti-FOXP3 FITC (PCH101), anti-CD8 APC-Cy7 (SK1) (eBioscience), anti-CD25 PECy7 (M-A251), anti-CD25 APC-Cy7 (M-A251), anti-CD127 PE (HIL-7R-M2), anti-CD27 FITC (M-T271), anti-CD70 PE (Ki-24), anti-CD3 PECy7 (SK7), anti-mouse CD45 FITC (30-F11) (BD Biosciences), anti-FOXP3 Alexa Fluor 647 (259D), anti-CD27 PECy7 (M-T271), anti-CD70 APC (113-16) (BioLegend) and anti-BCL-XL (7B2.5) (Abcam, UK) specific antibodies. Intracellular staining was performed using FOXP3 staining buffers (eBioscience). For staining, antibodies were used in a 1:45 dilution. Data were acquired using a FACS Canto II and analysed using FlowJo software (Treestar).

### *TSDR DNA* methylation analysis

Methylation at the Treg-Specific Demethylated Region (TSDR) was studied in CD27^+^CD70^−^ and CD27^−^CD70^+^ Treg cell subsets from matched donors sorted from CD4^+^CD25^+^CD127^−/low^ Tregs either freshly isolated or after 2 weeks of in vitro expansion. Methylation analysis was conducted by EpigenDx by pyrosequencing of bisulphite-modified DNA purified from frozen cells. Nine representative CpG residues in the TSDR were analysed using assay ADS783-FS2 for human *FOXP3*.

### In vitro suppression assays

CD3^+^CD25^−^ T responder cells (Tresp) were negatively isolated from PBMCs by incubation with CD25^+^ Microbeads followed by the positive selection of CD3^+^ T cells by CD3 Microbeads using the MACS system (Miltenyi Biotec). For monocyte-derived dendritic cell (mo-DC) generation, CD14^+^ monocytes were magnetic bead-isolated from PBMCs (Miltenyi Biotec) and cultured with 50U/ml IL-4 and 50 ng/ml GM-CSF in complete media supplemented with 10% FCS. After 5 days of incubation, mo-DCs were harvested and stored in liquid nitrogen for later use.

Variable numbers of Tregs were incubated with 2 × 10^4^ allogenic monocyte-derived dendritic cells (allo mo-DCs) as stimulators and 10 × 10^4^ autologous CD3^+^CD25^−^ T cells labeled with 1 µM VPD450 (562158, BD Biosciences) proliferation dye as responders (Tresp) in triplicate wells. Labeled Tresp cultured alone in the presence of allo mo-DCs were used as a negative control for suppression. Following 80–96 h of incubation, co-cultures were stained to distinguish CD8^+^ and CD4^+^ effector T cells, and VPD dilution was analysed by flow cytometry. Percentage of suppression was calculated by using division index of co-cultures containing Tregs, Tresp and DCs compare to division index of Tresp with DCs alone, according to the following formula: (1 − (div.index Treg + Tresp/div.index Tresp)) × 100. Data were analysed with FlowJo software.

### In vivo proliferation assay

BALB/c Rag2^−/−^cγ^−/−^ mice were maintained in individually-ventilated cages under specific pathogen-free in the Biomedical Service Unite of the University of Oxford (Oxford, UK). Female mice were aged between 8 and 12 weeks at the time of the experimental procedure. Mice received an intraperitoneal injection of 5 × 10^6^ cryopreserved and thawed PBMCs labeled with 1 µM VPD450 (562158, BD Biosciences) together with 1 × 10^6^ cryopreserved and thawed 4-week expanded CD27^+^CD70^−^ or CD27^−^CD70^+^ Tregs. Mice were injected with different Treg subsets or no Tregs in a blind way and allocated randomly in different cages. Cells were extracted by peritoneal lavage at day 4 after transfer, stained, and analysed by flow cytometry. VPD dilution was assessed to calculate proliferation index with FlowJo software. On analysis, lavage fluid was procured and analysed in a blinded fashion before unblinding at the final analysis.

### *CD70* co-stimulation test

CD3^+^CD25^−^ T responder cells (Tresp) were purified from PBMCs by MACS (Miltenyi Biotec) and labeled with 1uM VPD450 (562158, BD Biosciences). T cells were cultured in plates coated with 1 µg/ml anti-CD3 mAb (OKT3, BioLegend) and in the presence of in vitro stimulated Treg subsets. Proliferation of responder CD4^+^ and CD8^+^ T cells was calculated as division index using FlowJo software.

### Anti-CD27 and anti-CD70 mAbs for functional assays

Anti-CD70 blocking mAb (clone BU.69) was purchased from Abcam (UK) as mouse anti-human antibody or from Absolute Antibody Ltd as fully human Ab. Fully human anti-CD27 mAb and its Fc-mutated version were provided by Celldex Therapeutics. Secondary goat anti-human antibody for crosslinking was purchased from Abcam (UK). Isotype control antibodies were mouse IgG1 (BioLegend), fully human IgG1 (BioXcell, NH, USA) and human IgG1 Fc-silent (Absolute Antibody Ltd, UK). Where indicated, Tregs were pre-incubated with 20 µg/ml anti-CD70, anti-CD27 or control isotype antibodies for 1 h at 37 °C. Excess antibodies were washed out before assessing functionality of pre-incubated Tregs. Alternatively, 20 µg/ml anti-CD70, Fc-mutated anti-CD27 blocking mAbs or corresponding isotype antibody was added to the functional assay.

### *Bulk RNA*-sequencing analysis

RNA sequencing (RNA-seq) analysis was performed in 4 weeks expanded CD27^+^CD70^−^ and CD27^−^CD70^+^ Tregs as depicted in Fig. 3a for four independent donors. Before analysis, expanded cell products were re-sorted according to CD27 and CD70 expression to high purity. RNA was isolated from 300,000 CD27^+^CD70^−^ and CD27^−^CD70^+^ Tregs cells using RNeasy Plus Mini Kit (QIAGEN) according to the manufacturer’s protocol. mRNA capture and cDNA synthesis were performed using KAPA mRNA Hyper Kit for illumine platform (KAPA) according to the manufacturer’s protocol. After quality check using Qubit High-sensitivity kit (Life Technologies), 16 cycles of PCR were carried out for amplifying each library. Each experiment starting from RNA isolation was performed in triplicate using cells from four different donors. Amplified cDNA libraries were sequenced on an Illumina HiSeq4000 machine using paired end reads (2 × 150 bp) at Novogene, China. Forty million reads per sample were gained and aligned using STAR against the human genome (UCSC hg38). The non-adjusted read counts for each gene were assessed statistically for global differential expression between the specified populations using the edgeR package. Genes that are significant at a 1% false discovery rate (calculated using a Benajmini–Hochberg adjusted *p*-value) were considered differentially expressed between populations as described^93^. Gene ontology enrichment was assessed using the topGO package and pathway enrichment was analysed on the ReactomePA package. Sequencing data is available from the NCBI GEO database under accession numbers GSE129251.

### Single cell RNA sequencing reanalysis

Single cell RNA sequencing data presented in this manuscript are reanalyzed data from three blood donors presented in Trzupek et al.^68^. Detailed description of sorting strategy, library preparation, sequencing and data analysis and QC is presented in Trzupek et al.^68^. Briefly, CD4^+^ T cells enriched from PBMCs from one SLE patient, one T1D patient and one autoantibody negative healthy donor were stained with sorting antibodies and BD AbSeq antibodies (BD Biosciences) against 43 T-cell expressed targets, and sorted as CD127^low^CD25^hi^ Tregs, CD127^hi^ Teffs and CD127^low^ Teffs. Sorted subsets were barcoded with oligo-conjugated sample multiplexing tag antibodies (BD Biosciences) and mixed at 1:1:1 ratio to increase the relative distribution of less abundant Tregs and CD127^low^ Teffs. Cells were next loaded on a BD Rhapsody cartridge (BD Biosciences) and captured as single cells using the BD Rhapsody Express system according to manufacturer’s instructions. cDNA libraries were prepared using Human Immune Response primer panel (BD Biosciences) and sequenced using HiSeq 4000 sequencer (Illumina). Data analysis and QC was performed following the BD Biosciences Rhapsody pipeline (BD Biosciences) and the R package Seurat 3.0^94^, as previously described^68^. For the memory/effector-restricted T cell analysis presented here, the identified naive clusters (0, 1, and 2 in ref. ^68^) were excluded leading to the following number of remaining cells used in the analysis: SLE patient *n* = 5167, T1D patient *n* = 3154, healthy donor *n* = 2376. Uniform manifold approximation and projection (UMAP) was used for dimensionality reduction. The default number of used dimensions of PCA reduction was increased to 30 and the default resolution parameter value for clustering was increased to 1.4 to obtain more fine-grained set of clusters. Differential expression analysis was performed using a tailored hurdle model from MAST package^95^ used by Seurat.

### CRISPR/Cas9 genome editing

A third-generation self-inactivating “terminal” lentiviral vector expressing GFP and single guide RNA (sgRNA) was generated as described previously^96^. The vector incorporates guide sequences into the U3 region of the 3′LTR, thereby coupling transgene expression and CRISPR effects after subsequent Cas9 delivery by electroporation. Concentrated vector preparations were produced by transient transfection of 293T cells^97^. Ten days in vitro expanded-CD4^+^CD25^+^CD127^−/low^ Tregs were transduced with lentiviral vector at a MOI of 5, and CleanCap™ *Streptococcus pyogenes* Cas9 (spCas9) mRNA (L-7606 TriLink Biotechnologies San Diego, USA) was delivered by electroporation 3 days later at a concentration of 100 µg/ml. Lentiviral transduced Tregs that were not subjected to electroporation or that were electroporated in the absence of Cas9 mRNA were used as a control for genetic knockout (KO) effect. Successfully transduced Tregs were flow sorted based on GFP expression and expanded in vitro prior to additional analysis. Alternatively, sgRNA was complexed with spCas9 protein and delivered as ribonucleoprotein (RNP) by electroporation in 2 weeks in vitro expanded Tregs. Gene-specific sgRNAs were obtained from Integrated DNA Technologies, Inc. as CRISPR RNAs (crRNAs) and tracer RNA (tracrRNA). SpCas9 was purchased as Alt-R S.p. HiFi Cas9 Nuclease from Integrated DNA Technologies, Inc (cat: 1081061). RNP complexes were prepared according to the manufacturer’s instruction. Tregs electroporated with Cas9 without sgRNA were used as a control for KO effect.

Electroporation was performed in 100 µl tips using a protocol consisting of three pulses at 1600 V and 10 ms on the Neon Transfection System (MPK10025, Neon System Kit, ThermoFisher Scientific) in accordance with manufacturer’s instructions.

sgRNA sequences targeting CD70 were described previously^52^ or designed using Benchling software (https://benchling.com/crispr). sgRNA sequences were: 1_3: GCTACGTATCCATCGTGA, 2_3: GTACACATCCAGGTGACGC, 3_3: GCAGGCTGATGCTACGGG and 1_1: TCACCAAGCCCGCGACCAAT.

KO efficiency was calculated as percentage of CD70^+^ cells in Tregs electroporated with Cas9 mRNA or RNPs compared to Tregs that were not electroporated or that were electroporated in the absence of Cas9 mRNA or sgRNA according to the following formula: (100 − (%positive cells in KO-Tregs * 100/%positive cells in control-Tregs)). In addition, genetic modifications at the *CD70* locus were analyzed using ICE protocols (https://ice.synthego.com/). Genomic DNA extraction was performed using DNeasy Blood and Tissue Kit (69504, Qiagen, Hilden, Germany) and amplification of 700 bp around sites of predicted Cas9 scission was performed by PCR. Primers were 1_3 forward: CACCGGCTACGTATCCATCGTGA, 1_3 reverse: AAACTCACGATGGATACGTAGCC, 3_3 forward: CACCGCAGGCTGATGCTACGGG and 3_3 reverse: AAACCCCGTAGCATCAGCCTGC. PCR products were sequenced and analyzed using ICE algorithm.

### Study approvals

All mouse experiments were performed using protocols approved by the Committee on Animal Care and Ethical Review at the University of Oxford and in accordance with the UK Animals (Scientific Procedures) Act 1986 and under PPL number P8869535A. Experiments using donated human cells were performed with ethical approval from the Oxfordshire Research Ethics Committee (REC B), study number 07/H0605/130. Single cell sequencing experiments were performed with ethical approval from Peterborough and Fenland Research Ethics Committee (05/Q0106/20) for SLE sample and the Royal Free Hospital & Medical School Research Ethics Committee; REC (08/H0720/25; D-GAP study) for T1D and HD samples. Informed consent was obtained from all subjects.

### Statistics and reproducibility

Graphs were produced and statistical analyses performed using Prism version 5 or 7 (GraphPad Software, San Diego, CA, USA). Statistical significance was determined as indicated in the figure legends. Non-parametric tests were selected. Unpaired tests were performed to compare between cell subsets, and paired tests were used when comparing a specific cell subset over time or upon treatment with monoclonal antibodies. SD is represented in graphs showing replicates from one representative donor or in graphs with data from multiple donors, when each data point displays the data of one donor. SEM is used in graphs with data from multiple donors, when each data point represents a mean of multiple replicates for each donor. Functional assays were set up with, at least, three replicates for each sample. All source data underlying the graphs presented in the main figures are included in Supplementary Data 1 file.

### Reporting summary

Further information on research design is available in the Nature Research Reporting Summary linked to this article.

## Supplementary information

Supplementary Information

Description of Additional Supplementary Files

Supplementary Data 1.

Supplementary Data 2.

Supplementary Data 3.

Supplementary Data 4.

Reporting Summary

## Data Availability

The authors declare that all data supporting the findings of this study are available within the paper and its supplementary information files. Please contact the corresponding author (J.H.) for access of raw data, which is stored electronically, and will be made available upon reasonable requests. Sequencing data is available from the NCBI GEO database under accession numbers GSE129251.
