# Supplementary material for: CD70 expression determines the therapeutic efficacy of expanded human regulatory T cells
Source: Commun Biol. 2020 Jul 14;3:375. doi: 10.1038/s42003-020-1097-8 (PMC7360768; doi:10.1038/s42003-020-1097-8)
Supplement: Supplementary file 1 — Supplementary Information [file 42003_2020_1097_MOESM1_ESM.pdf]

CD70 expression determines the therapeutic efficacy of expanded human regulatory T cells

Supplementary figures

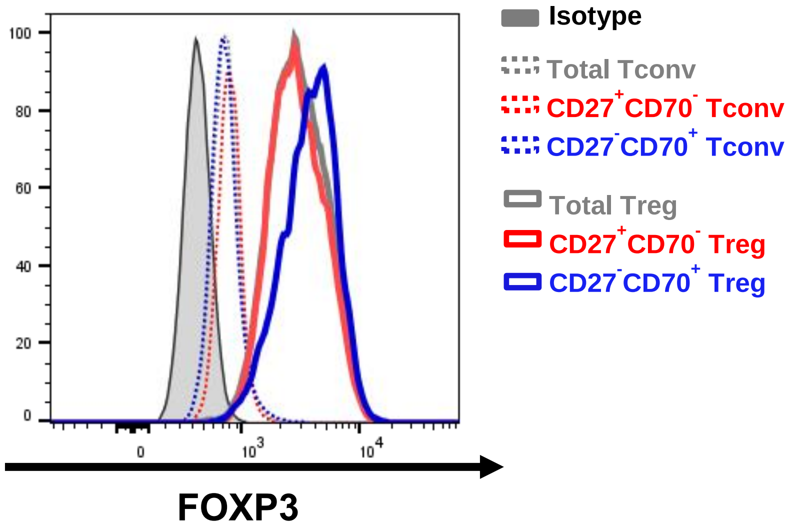

Supplementary Figure 1. Higher FOXP3 expression in *in vitro* expanded CD27$^-$CD70$^+$ Tregs compared to CD27$^-$CD70$^+$ conventional T cells. FOXP3 expression was analyzed in 2-week expanded total Tregs or CD4$^+$ conventional T cells (Tregs isolated as CD4$^+$CD25$^+$CD127$^-$ cells and Tconv isolated as CD4$^+$CD25$^-$ cells and expanded *in vitro* with anti-CD3/anti-CD28 coated beads in the presence of rhIL-2), and gated on CD27/CD70 Treg populations.

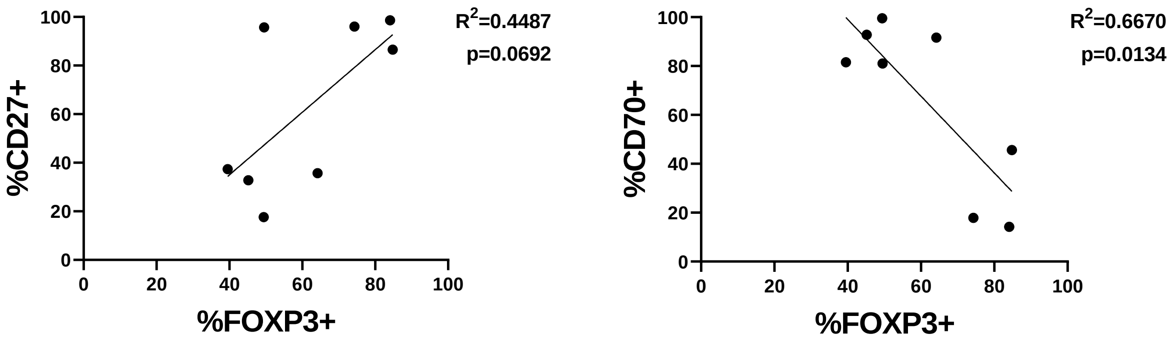

Supplementary Figure 2. Correlation between FOXP3 and CD27 or CD70 expression in Tregs expanded *in vitro* for 4 weeks. Percentage expression was determined by flow cytometry. Each dot represents a separate blood donor.

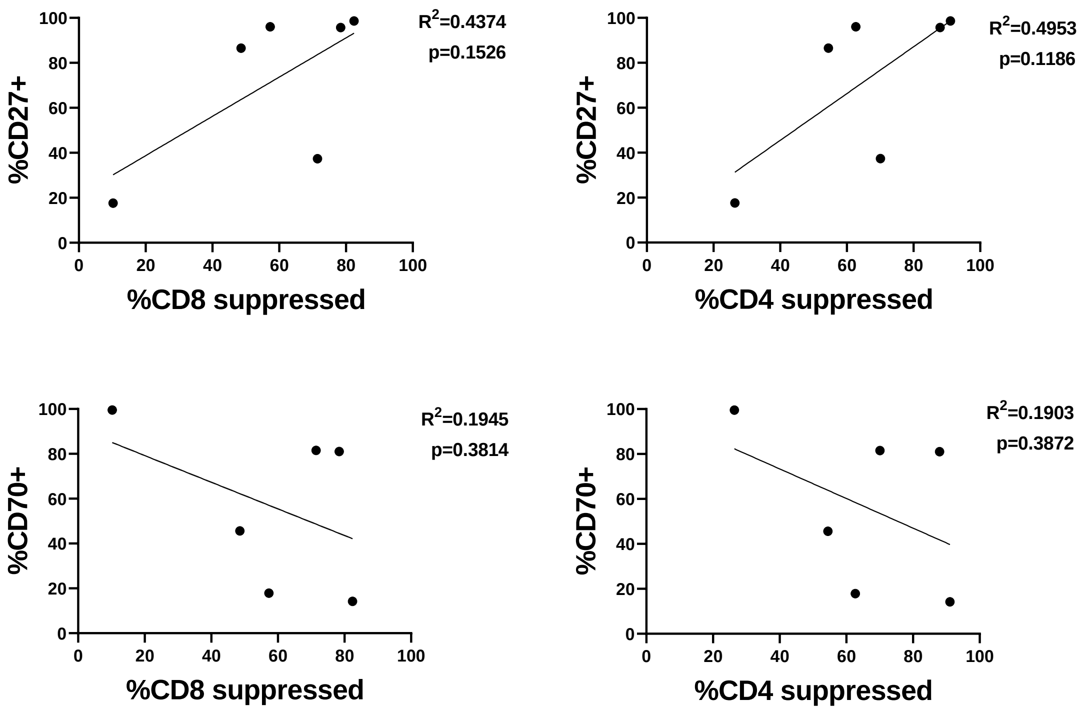

Supplementary Figure 3. Correlation between suppression of $CD4^+$ or $CD8^+$ T cell proliferation and CD27 or CD70 expression in Tregs expanded *in vitro* for 4 weeks. T cell suppression by Tregs was assessed using an *in vitro* VPD-dilution suppression assay. Each dot represents a separate blood donor, with each dot being a mean of 3 repeats of each suppression assay.

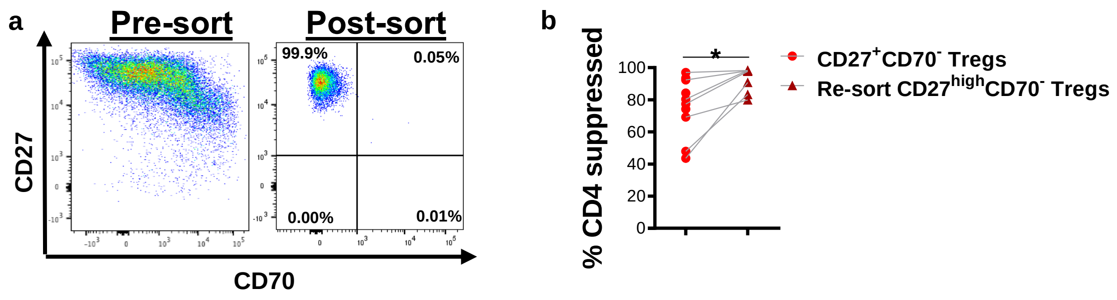

Supplementary Figure 4. The CD27^{high}CD70^{-} phenotype identifies Tregs with potent suppressive capacity. (a) CD27/CD70 expression analyzed in 4 weeks expanded Tregs (sorted as total Tregs, expanded for 2 weeks, sorted for CD27^{+}CD70^{-} Tregs and then expanded for 2 weeks), (left panel) prior or (right panel) after re-sorting for CD27^{+}CD70^{-} Tregs. **(b)** Suppressive capacity of re-sorted CD27^{high}CD70^{-} Tregs within total CD27^{+}CD70^{-} expanded cells. Treg suppressive activity was assessed *in vitro*. Each data point represents the mean of 3 replicate wells for each of 7 independent donors. Data were analyzed using a Wilcoxon matched-pairs test.

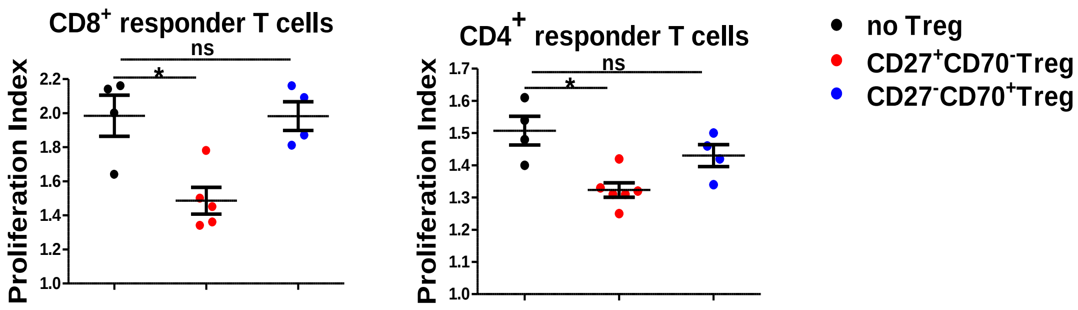

Supplementary Figure 5. CD27^{-}CD70^{+} Tregs have defective suppressive activity *in vivo*. Suppressive capacity of CD27/CD70 Treg subsets was assessed *in vivo* as described in Figure 3E. The proliferation index of PBMC injected with or without CD27^{+}CD70^{-} or CD27^{-}CD70^{+} Tregs is shown. Results from a second cell donor are shown (4-5 mice per group per experiment). Data were analyzed using a Kruskal-Wallis test with Dunn's post-test for multiple comparisons and are represented as mean ±SD. ns= non-significant, *p<0.05.

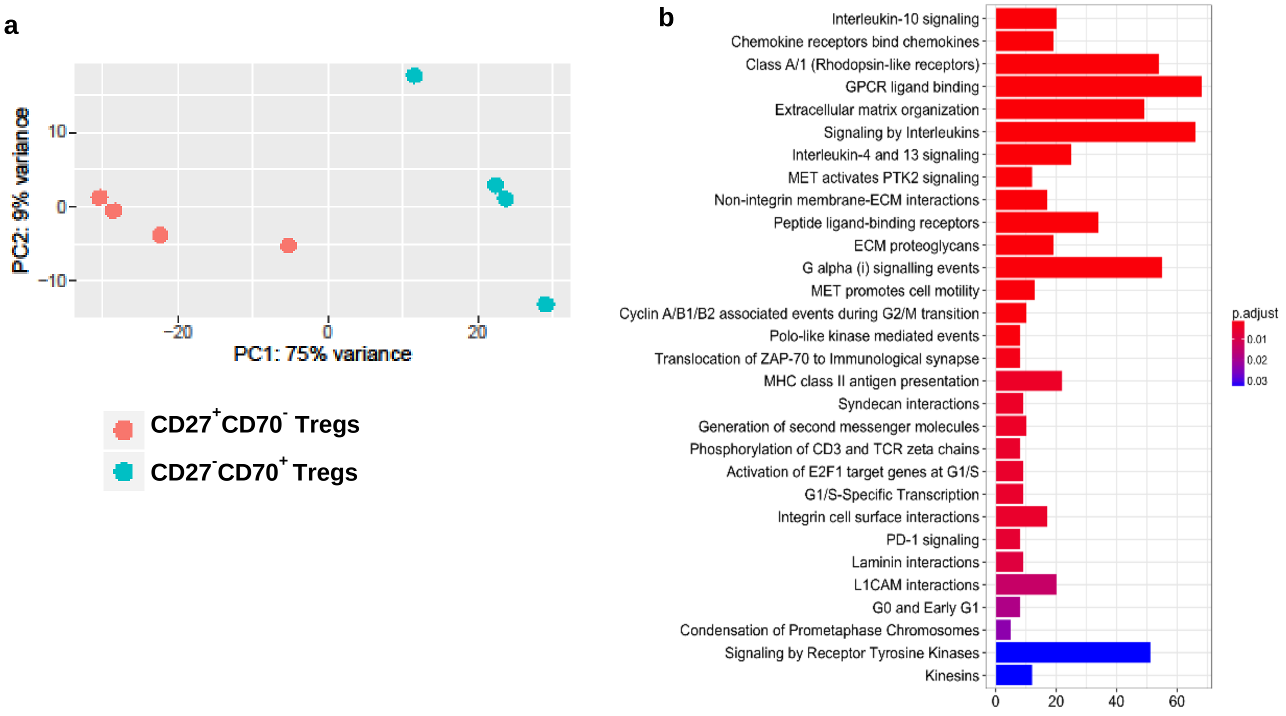

Supplementary Figure 6. Principal component analysis of in the RNA-seq analysis of CD27/CD70 Treg subsets. (a) Principal component analysis of RNA-seq data from CD27^{+}CD70^{-} and CD27^{-}CD70^{+} Treg subsets isolated from 4 independent donors. Each sample was run in triplicate. Data were analyzed using the edgeR package. **(b)** Gene ontology enrichment plot obtained using the topGO R package. x axis shows the number of genes for each gene ontology term (y axis). The number of genes within each pathway that are differentially expressed (which could be up or down) between the two subsets is represented. Associated *p* values are shown in a color code as a heatmap.

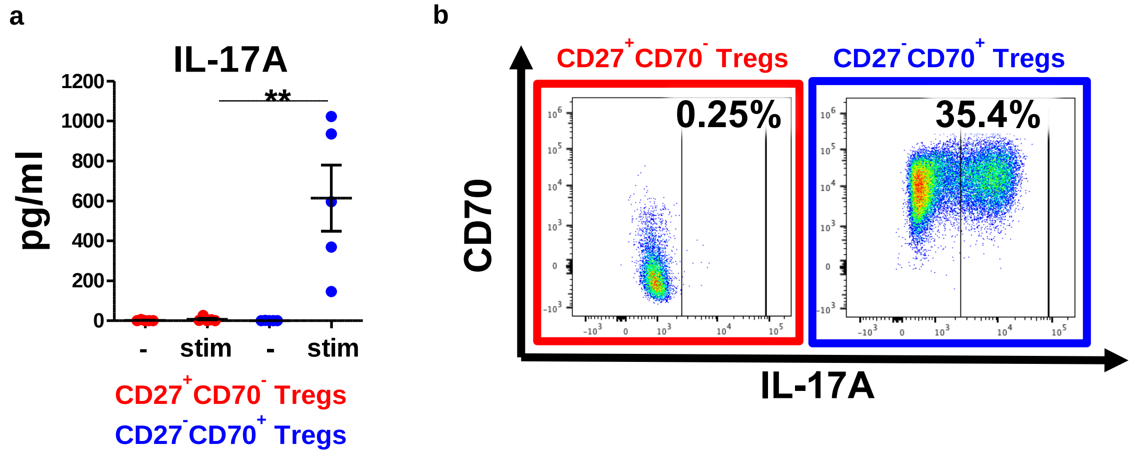

Supplementary Figure 7. CD27^{-}CD70^{+} Tregs, but not CD27^{+}CD70^{-} Tregs, express IL-17A. **(a)** 4 weeks expanded CD27^{+}CD70^{-} (red) or CD27^{-}CD70^{+} (blue) Tregs were rested for 2 days prior to a 72h-stimulation in the presence of rhIL-2 (250 U/ml) with or without anti-CD3/anti-CD28 coated beads (1 bead: 5 cells). A cytokine bead array was performed on supernatants. (-) denotes unstimulated cells and (stim) represents Tregs that have been stimulated with anti-CD3/anti-CD28-coated beads. n=5 independent donors (each data point is the average of 3 replicate wells in the bead array assay of each donor). Statistical significance comparing stimulated Treg subsets was assessed using a Mann-Whitney test. Data are represented as mean ± SEM. **(b)** 4 week-expanded, and 5 day-rested CD27^{+}CD70^{-} and CD27^{-}CD70^{+} Tregs were stimulated with phorbol myristate acetate (PMA) (100ng/ml) and ionomycin (1μg/ml) in the presence of Golgi protein inhibitor for 5 hours. Intracellular IL-17A expression was analyzed by flow cytometry.

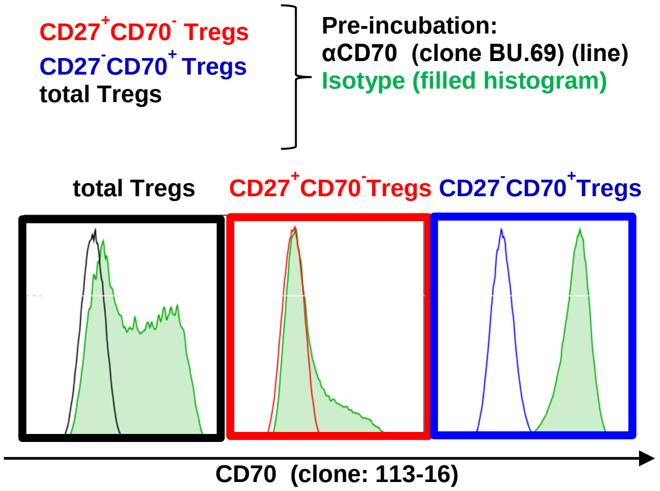

Supplementary Figure 8. Anti-CD70 blocking mAb effectively binds to CD70 expressed on Tregs. Expanded Treg subsets were pre-incubated with anti-CD70 blocking mAb or control isotype antibody and effective antibody binding was confirmed by flow cytometry using an anti-CD70-fluorochrome mAb that is sterically hindered by anti-CD70 blocking mAb. CD70 expression is shown by green histogram on isotype-incubated Tregs and by solid lines on anti-CD70-incubated Tregs.

a

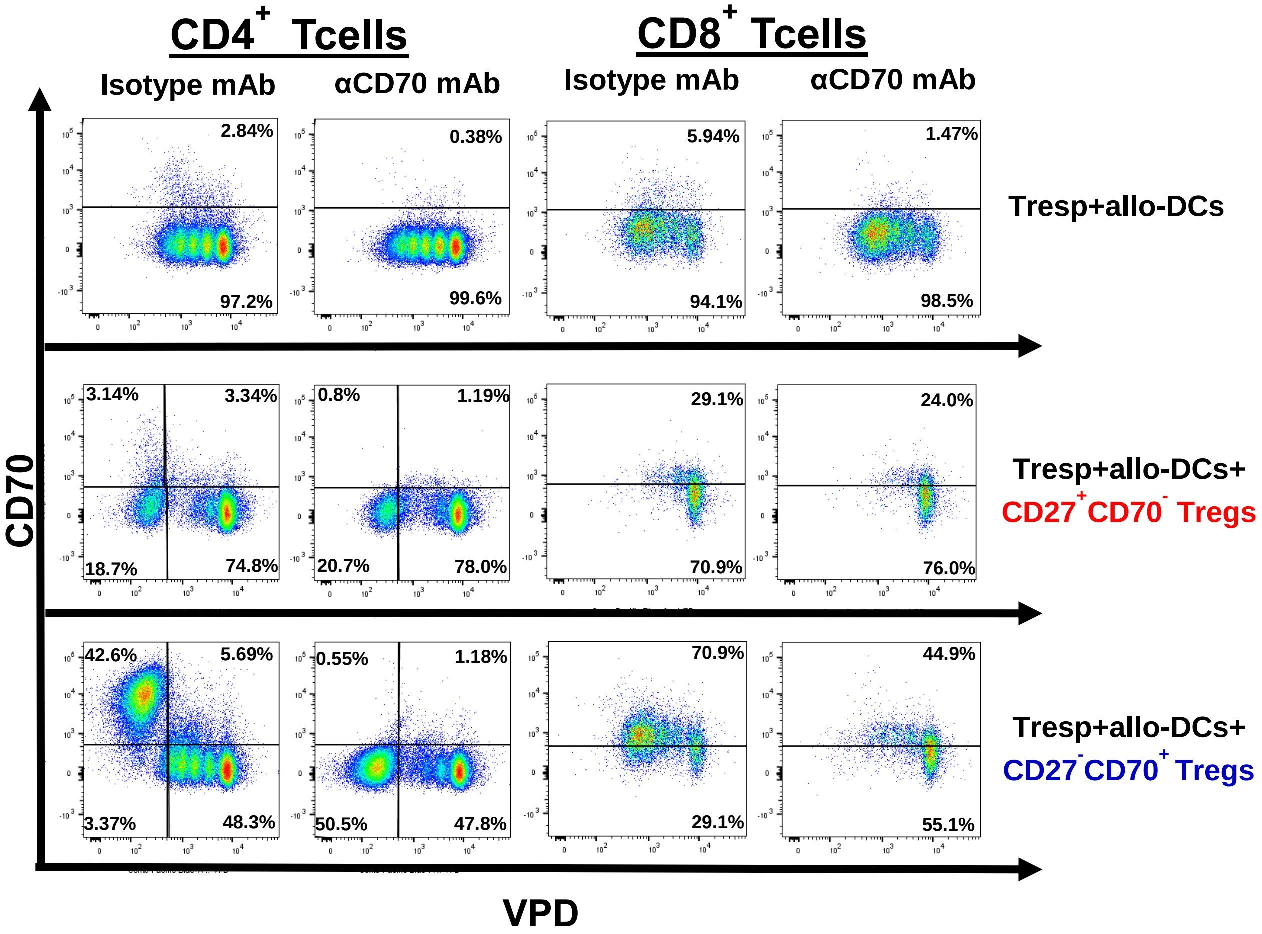

b

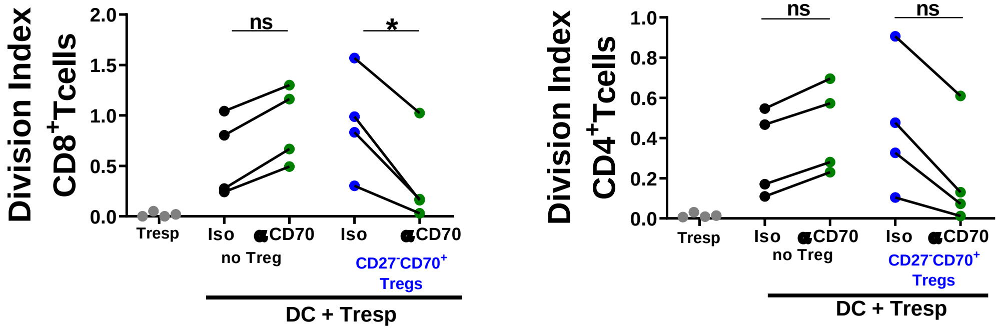

Supplementary Figure 9. CD70 blockade does not impair proliferation of T cells activated with allo mo-DCs. CD3$^+$CD25$^-$ responder T cells (Tresp) were stained with VPD and stimulated with allo mo-DCs in the absence of Tregs (black) or in the presence of CD27$^+$CD70$^-$ Tregs (red)

or $CD27^{-}CD70^{+}$ Tregs (blue). Isotype control antibody or anti-CD70 mAb (20μg/ml) was added to the co-culture assays. **(a)** VPD and CD70 expression was assessed in $CD4^{+}$ T cells and $CD8^{+}$ T cells at the end of the co-culture when isotype or anti-CD70 mAb was added to the assay. **(b)** Proliferation of Tresp was assessed by VPD dilution and measured by flow cytometry. Proliferation of responder $CD8^{+}$ or $CD4^{+}$ T cells is represented as division index. Each dot represents an independent cell donor (n=4 independent cell donors). The average of 3 replicate wells in the suppression assay of each donor is shown. Groups were compared using a Friedman test with Dunn's post-test for multiple comparisons. ns= non-significant, *$p<0.05$.

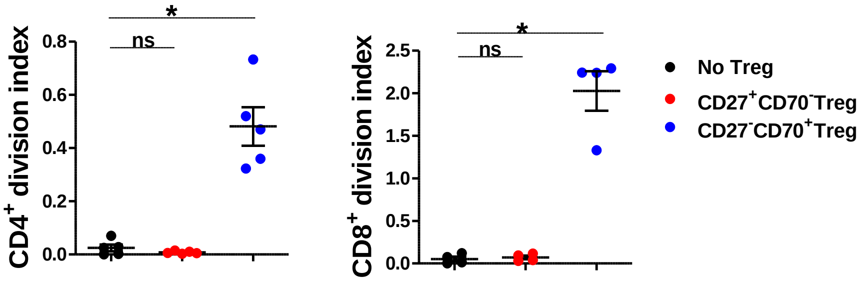

Supplementary Figure 10. CD27$^-$CD70$^+$ but not CD27$^+$CD70$^-$ Tregs provide co-stimulation to conventional T cells. VPD-labelled CD25$^-$CD3$^+$ T cells (Tresp) were co-cultured with 4 week-expanded CD27$^+$CD70$^-$ Tregs or CD27$^-$CD70$^+$ Tregs in wells containing plate-bound anti-CD3 mAb (1µg/ml). Data show proliferation of CD4$^+$ and CD8$^+$ Tresp, represented as division index. Each data point represents the mean of 3 replicate wells for each of 5 independent cell donors. Data were analyzed using a Kruskal-Wallis test with Dunn's post-test for multiple comparisons. ns= non-significant, *p<0.05.

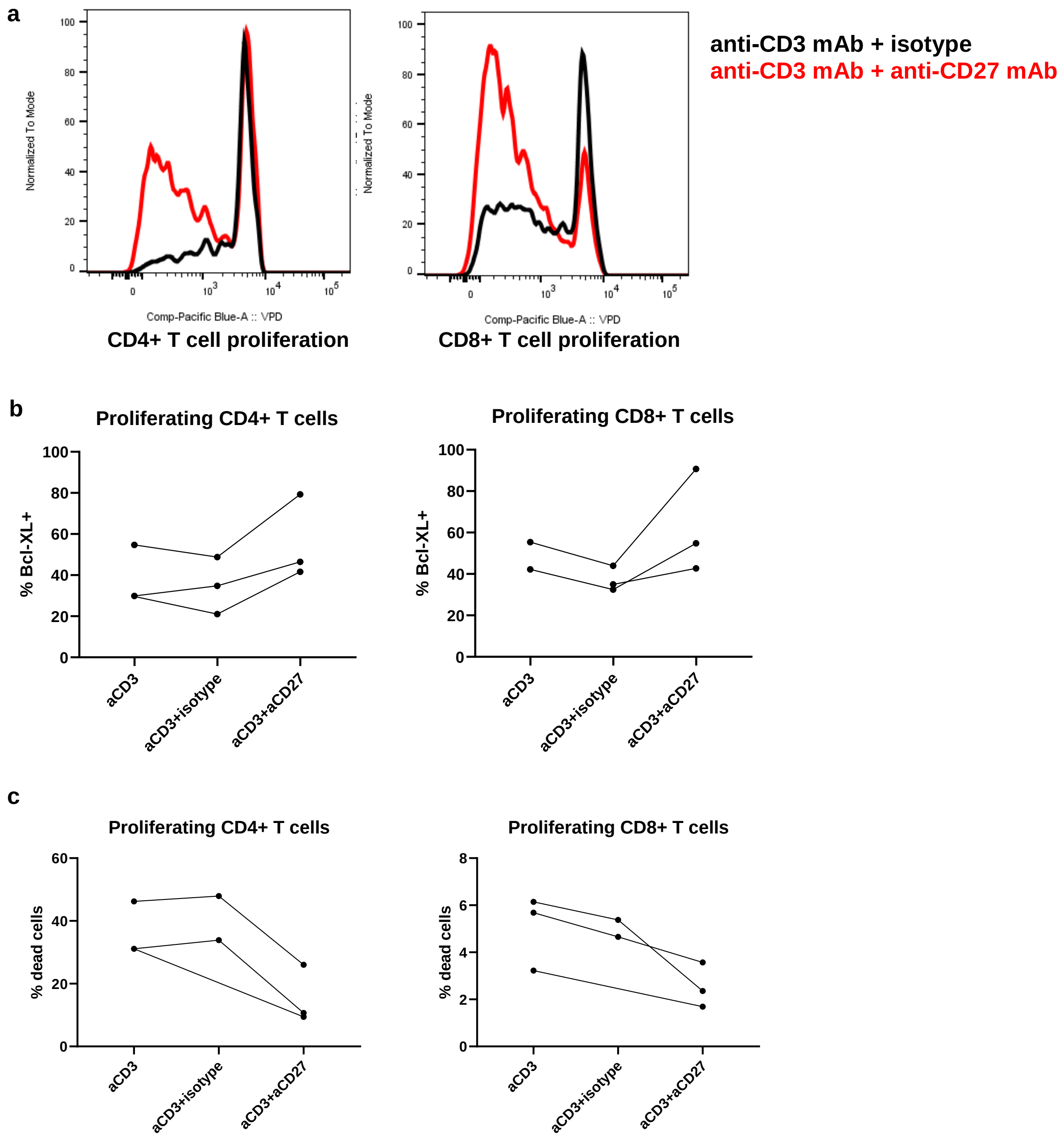

Supplementary Figure 11. CD27 stimulation activates survival signals in conventional T cells. VPD-labelled CD3+ T cells were stimulated in wells containing plate bound anti-CD3 mAb (1μg/ml) with or without plate bound isotype hIgG1 mAb or anti-CD27 agonist mAb (20μg/ml). Cells were cultured for 5 days. **(a)** Histogram plots represent CD4+ or CD8+ T cell proliferation

under different stimulation conditions. Cell proliferation is measured by VPD dilution. One representative donor out of 3 is shown. Black: anti-CD3 mAb and isotype mAb stimulation, red: anti-CD3 mAb and anti-CD27 stimulation. **(b)** Bcl-XL was studied within proliferating $CD4^+$ or $CD8^+$ T cells (gated as cells that diluted VPD). **(c)** Percentage of dead cells in $CD4^+$ and $CD8^+$ T cells was studied by staining with 7AAD. Each dot represents a separate blood donor, with each dot being a mean of 3-4 repeats of each experimental condition.

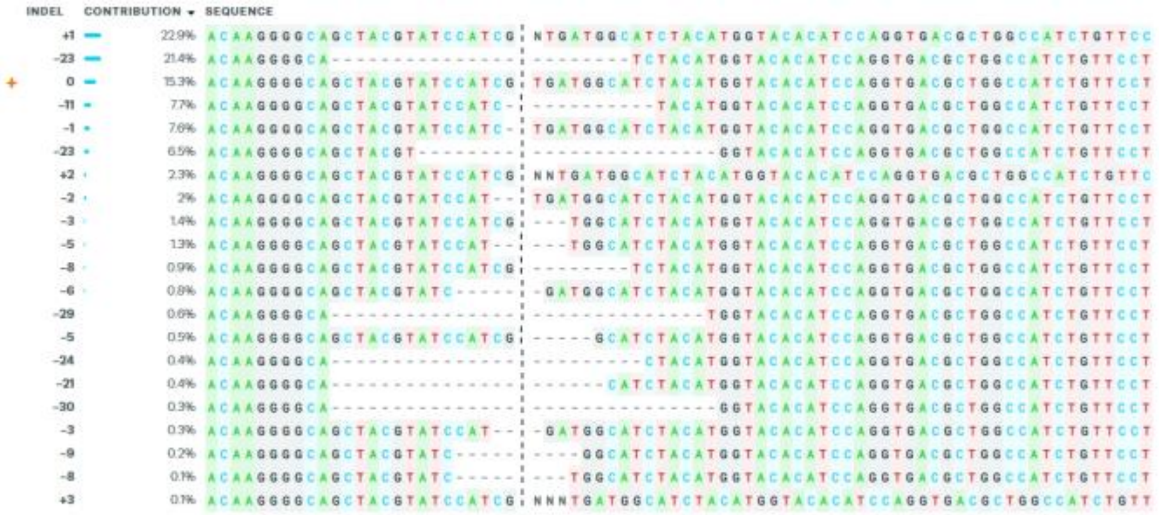

Supplementary Figure 12. Molecular signature of the CD70 knockout effect. Table showing all the sequences within the CD70-KO Treg population. For each sequence, the number of nucleotides inserted or deleted and the relative abundance of each indel within the edited population is represented on the left. + indicates the wild type sequence, and its prevalence in the edited population. The vertical black dotted line shows the cut site. Base pair deletions are indicated by dashes, and insertions are indicated by “N” bases. The effect of sgRNA 1_3 for 1 representative donor is shown. Analysis was performed using ICE algorithm.

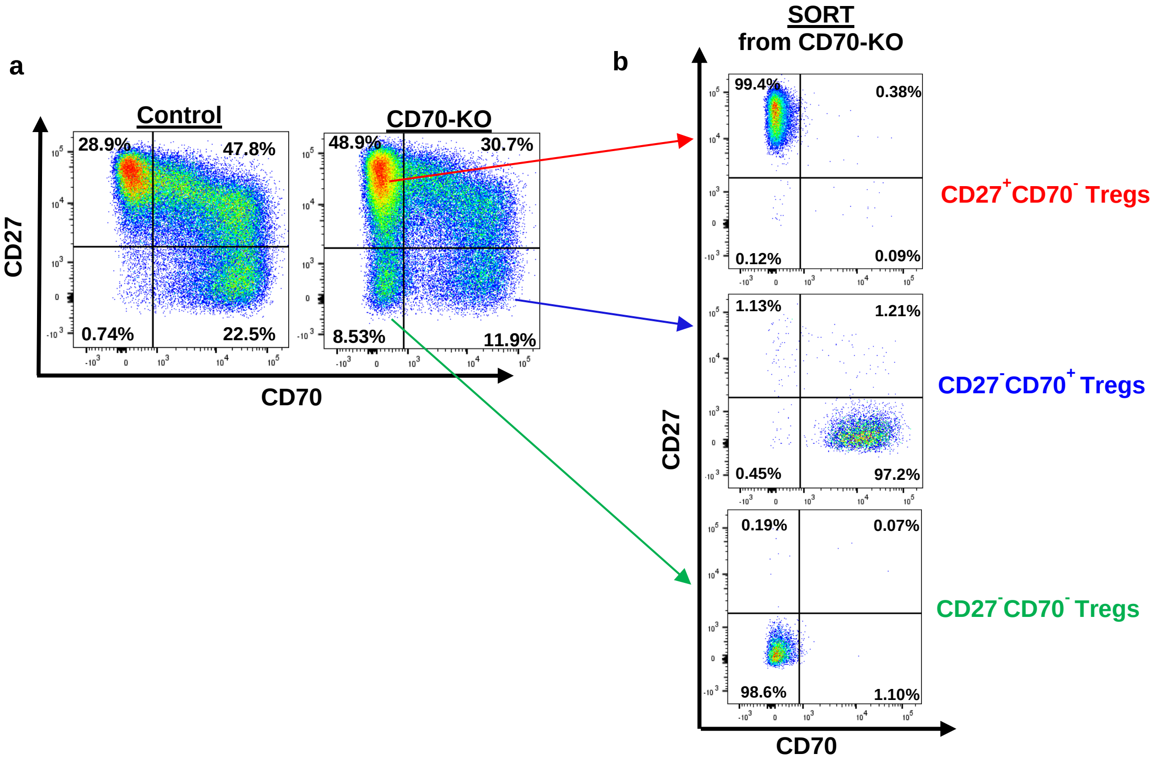

Supplementary Figure 13. Gating strategy for the isolation of Treg populations based on CD27 and CD70 expression from CD70-KO Treg cell products. (A) CD27 and CD70 expression measured on 29 days *in vitro* expanded Tregs that were electroporated with sgRNA 1_1 coupled to spCas9 proteins as ribonucleoprotein (CD70-KO) or with spCas9 protein without sgRNA (control). **(B)** Plots showing purity of $CD27^{+}CD70^{-}$, $CD27^{-}CD70^{+}$ and $CD27^{-}CD70^{-}$ Treg subsets that were FACS-sorted from CD70-KO cell products.

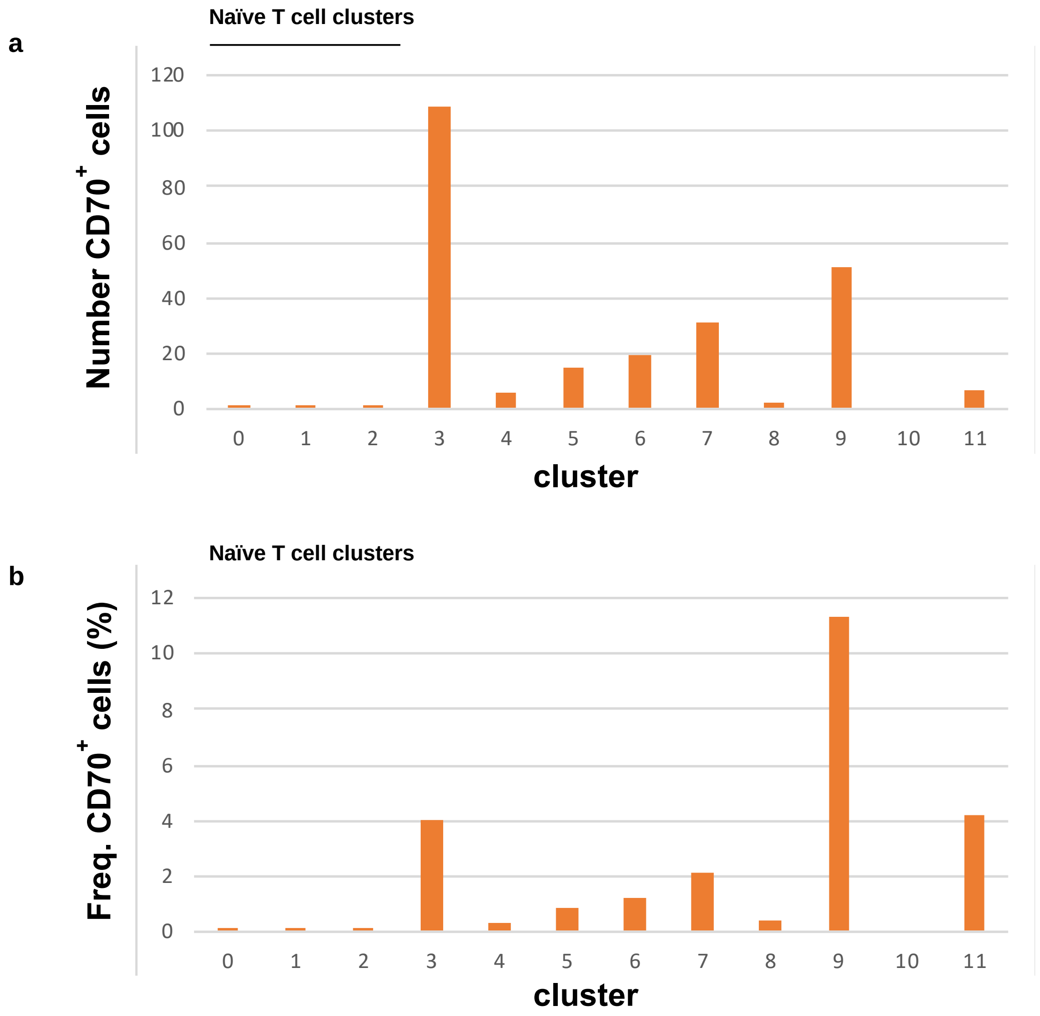

Supplementary Figure 14. Expression of CD70 within human peripheral blood primary CD4$^+$ T cell clusters defined in Trzupek *et al.* [1] using combined targeted scRNA-seq and protein expression analysis. Number **(a)** and frequency **(b)** of CD70$^+$ cells within each of the subsets is shown. Naive T cell clusters (0, 1 and 2) are marked.

a

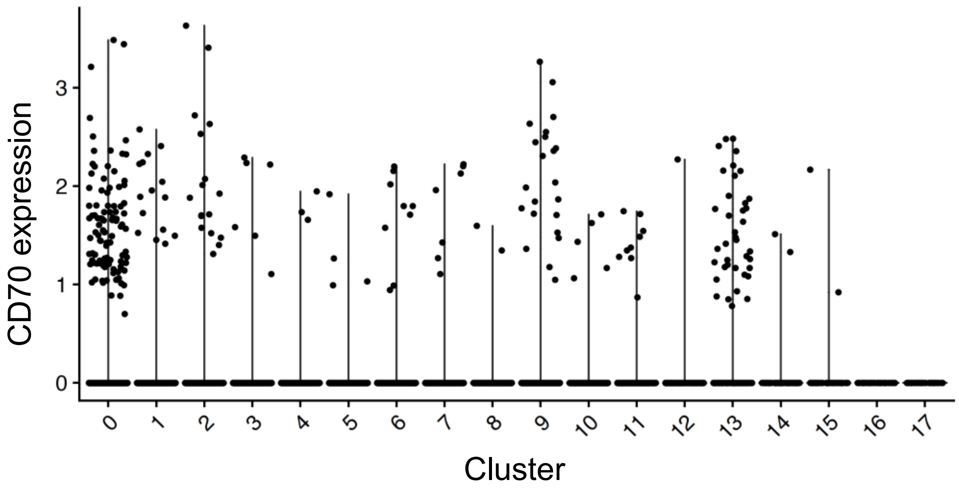

b

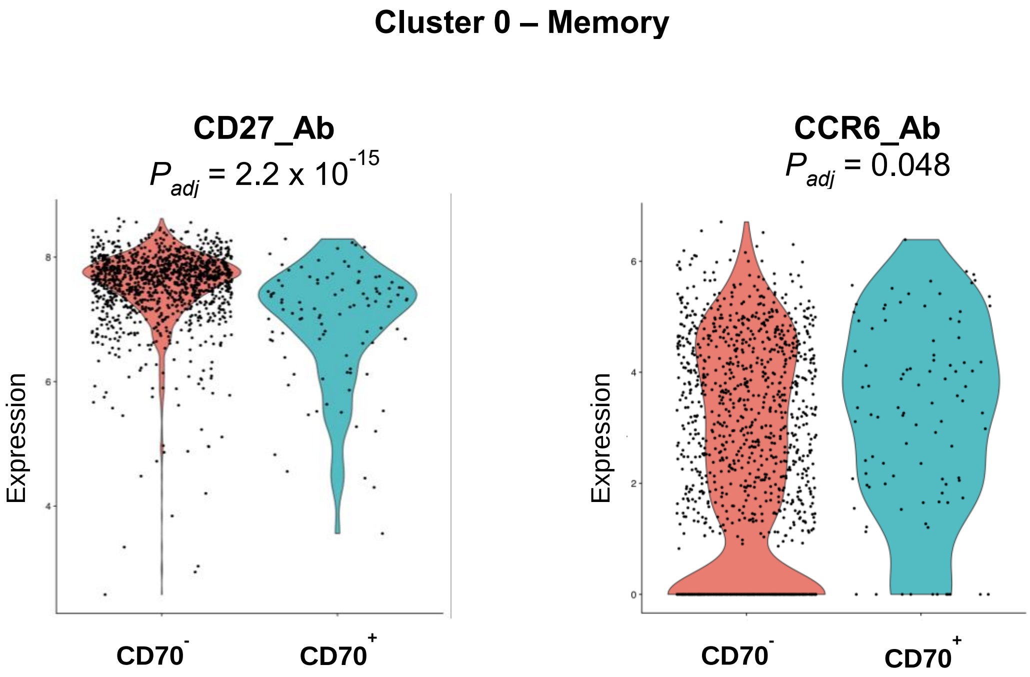

Supplementary Figure 15. Human peripheral blood primary CD4^{+} CD70 mRNA expression and correlation analysis using combined targeted scRNA-seq and protein expression analysis. (a) Normalised expression level of CD70 mRNA within each single-cell contained in the UMAP clusters defined in Figure 7. **(b)** Violin plots depicting the distribution of normalised expression of CD27 and CCR6 at the protein level between CD70^{+} and CD70^{-} cells within Treg Cluster 0.

Supplementary references

Uncategorized References

1 Trzupek, D. *et al.* Simultaneous mRNA and protein quantification at the single-cell level delineates trajectories of CD4+ T-cell differentiation. *bioRxiv*, doi:https://doi.org/10.1101/706275 (2019).
