# Supplementary material for: CD70 expression determines the therapeutic efficacy of expanded human regulatory T cells
Source: Commun Biol. 2020 Jul 14;3:375. doi: 10.1038/s42003-020-1097-8 (PMC7360768; doi:10.1038/s42003-020-1097-8)
Supplement: Supplementary file 2 — Description of Additional Supplementary Files [file 42003_2020_1097_MOESM2_ESM.pdf]

Descriptions of Additional Supplementary Files

Supplementary Data 1. Source data for the plots of Figures 1-6.

Supplementary Data 2. Results of DNA methylation analysis of the Treg-specific Demethylation Region (TSDR) of unstimulated or 2 weeks-*in vitro* expanded $CD27^{+}CD70^{-}$ and $CD27^{-}CD70^{+}$ Treg populations.

Supplementary Data 3. List of genes that were differentially expressed between 4 weeks-*in vitro* expanded $CD27^{+}CD70^{-}$ and $CD27^{-}CD70^{+}$ Treg populations.

Supplementary Data 4. List of genes that were upregulated in Treg or Tconv cells in Ferraro *et al*. (ref 65) and $CD27^{+}CD70^{-}$ or $CD27^{-}CD70^{+}$ Treg populations.

Executive Assistant and Project Officer: Monica Dolton
Tel: + 44 (0) 1865 612273 Email: monica.dolton@nds.ox.ac.uk
