# Supplementary material for: CD70 expression determines the therapeutic efficacy of expanded human regulatory T cells
Source: Commun Biol. 2020 Jul 14;3:375. doi: 10.1038/s42003-020-1097-8 (PMC7360768; doi:10.1038/s42003-020-1097-8)
Supplement: Supplementary file 7 — Reporting Summary [file 42003_2020_1097_MOESM7_ESM.pdf]

nature research

Corresponding author(s): Joanna Hester

Last updated by author(s): 10 April 2020

Reporting Summary

Nature Research wishes to improve the reproducibility of the work that we publish. This form provides structure for consistency and transparency in reporting. For further information on Nature Research policies, see Authors & Referees and the Editorial Policy Checklist.

Statistics

For all statistical analyses, confirm that the following items are present in the figure legend, table legend, main text, or Methods section.

n/a	Confirmed	
☐	☒	The exact sample size (*n*) for each experimental group/condition, given as a discrete number and unit of measurement
☐	☒	A statement on whether measurements were taken from distinct samples or whether the same sample was measured repeatedly
☐	☒	The statistical test(s) used AND whether they are one- or two-sided<br>*Only common tests should be described solely by name; describe more complex techniques in the Methods section.*
☐	☒	A description of all covariates tested
☐	☒	A description of any assumptions or corrections, such as tests of normality and adjustment for multiple comparisons
☐	☒	A full description of the statistical parameters including central tendency (e.g. means) or other basic estimates (e.g. regression coefficient) AND variation (e.g. standard deviation) or associated estimates of uncertainty (e.g. confidence intervals)
☐	☒	For null hypothesis testing, the test statistic (e.g. *F*, *t*, *r*) with confidence intervals, effect sizes, degrees of freedom and *P* value noted<br>*Give P values as exact values whenever suitable.*
☒	☐	For Bayesian analysis, information on the choice of priors and Markov chain Monte Carlo settings
☐	☒	For hierarchical and complex designs, identification of the appropriate level for tests and full reporting of outcomes
☒	☐	Estimates of effect sizes (e.g. Cohen's *d*, Pearson's *r*), indicating how they were calculated

Our web collection on statistics for biologists contains articles on many of the points above.

Software and code

Policy information about availability of computer code

Data collection	Flow cytometric data was acquired using a FACS Canto II. BD Rhapsody Express system (BD Biosciences) was used for single cell experiments. HiSeq 4000 sequencer (Illumina) was used for sequencing.
Data analysis	FlowJo software (Treestar) was use for flow cytometric data analysis. Graphs were produced and statistical analyses performed using Prism version 5 or 7 (GraphPad Software, San Diego, CA, USA). R software was used for RNA sequencing analysis. Specifically, the STAR, edgeR, topGO and ReactomePA packages were used for bulk sequencing analysis and the BD Biosciences Rhapsody pipeline and the R package Seurat 3.0 were used for single cell sequencing analysis.

For manuscripts utilizing custom algorithms or software that are central to the research but not yet described in published literature, software must be made available to editors/reviewers. We strongly encourage code deposition in a community repository (e.g. GitHub). See the Nature Research guidelines for submitting code & software for further information.

Data

Policy information about availability of data

All manuscripts must include a data availability statement. This statement should provide the following information, where applicable:

- Accession codes, unique identifiers, or web links for publicly available datasets
- A list of figures that have associated raw data
- A description of any restrictions on data availability

Bulk RNA-sequencing data is available from the NCBI GEO database under accession numbers GSE129251. Single cell RNA sequencing data are re-analysed data from 3 blood donors presented previously in Trzupek et al.

Field-specific reporting

Please select the one below that is the best fit for your research. If you are not sure, read the appropriate sections before making your selection.

☒ Life sciences ☐ Behavioural & social sciences ☐ Ecological, evolutionary & environmental sciences

For a reference copy of the document with all sections, see nature.com/documents/nr-reporting-summary-flat.pdf

Life sciences study design

All studies must disclose on these points even when the disclosure is negative.

Sample size	For animal studies sample sizes were chosen based on previous experience.
Data exclusions	No data have been excluded from the analysis.
Replication	For in vitro assays, assays were repeated in triplicate and across multiple donors, as indincated in figure legends. All replicates are considered in the study.
Randomization	For animal studies, mice were randomized across and within cages to different experimental groups.
Blinding	For animal studies, mice were injected with different Treg subsets or no Tregs in a blind way and allocated randomly in different cages. On analysis, lavage fluid was procured and analysed in a blinded fashion before unblinding at the final analysis.

Reporting for specific materials, systems and methods

We require information from authors about some types of materials, experimental systems and methods used in many studies. Here, indicate whether each material, system or method listed is relevant to your study. If you are not sure if a list item applies to your research, read the appropriate section before selecting a response.

Materials & experimental systems

n/a	Involved in the study	
☐	☒	Antibodies
☒	☐	Eukaryotic cell lines
☒	☐	Palaeontology
☐	☒	Animals and other organisms
☐	☒	Human research participants
☒	☐	Clinical data

Methods

n/a	Involved in the study	
☒	☐	ChIP-seq
☐	☒	Flow cytometry
☒	☐	MRI-based neuroimaging

Antibodies

Antibodies used	Flow cytometry antibodies used:<br>anti-CD4 PE-eFluor 647 (RPA-T4) , anti-CD27 eFluor 450 (O323), anti-FOXP3 FITC (PCH101), anti-CD8 APC-Cy7 (SK1), anti-CD25 PECy7 (M-A251), anti-CD25 APC-Cy7 (M-A251), anti-CD127 PE (HIL-7R-M2), anti-CD27 FITC (M-T271), anti-CD70 PE (Ki-24), anti-CD3 PECy7 (SK7), anti-mouse CD45 FITC (30-F11) (all BD Biosciences)<br>anti-FOXP3 Alexa Fluor 647 (259D), anti-CD27 PECy7 (M-T271) and anti-CD70 APC (113-16) (all BioLegend)<br>anti-BCL-XL (7B2.5) (Abcam)<br>Monoclonal antibodies used in vitro:<br>anti-CD3 mAb (OKT3, BioLegend)<br>anti-CD70 blocking mAb (clone BU.69) was purchased from Abcam (UK) as mouse anti-human antibody or from Absolute Antibody Ltd as fully human Ab fully human anti-CD27 mAb and its Fc-mutated version were provided by Celldex Therapeutics secondary goat anti-human antibody for crosslinking was purchased from Abcam (UK)<br>Isotype control antibodies were mouse IgG1 (BioLegend), fully human IgG1 (BioXcell, NH, USA) and human IgG1 Fc-silent (Absolute Antibody Ltd, UK)
Validation	Antibodies were validated by their respective manufacturers.

Animals and other organisms

Policy information about studies involving animals; ARRIVE guidelines recommended for reporting animal research

Laboratory animals	Species: Mouse. Strain: BALB/c Rag2-/-cy-/-. Gender: females. Age: 8-12 weeks at time of first experimental procedure.

Wild animals	This study did not involve wild animals
Field-collected samples	This study did not involve samples collected from the field
Ethics oversight	All mouse experiments were performed using protocols approved by the Committee on Animal Care and Ethical Review at the University of Oxford and in accordance with the UK Animals (Scientific Procedures) Act 1986 and under PPL number P8869535A.

Note that full information on the approval of the study protocol must also be provided in the manuscript.

Human research participants

Policy information about studies involving human research participants

Population characteristics	Samples from healthy blood donors were randomly allocated to the study and no data on age, sex or any other population characteristic is available.
Recruitment	Healthy blood donors were recruited for research while donating blood for clinical purposes. No specific strategy was employed for recruitment.
Ethics oversight	Experiments using donated human cells were performed with ethical approval from the Oxfordshire Research Ethics Committee. Single cell sequencing experiments were performed with ethical approval from Peterborough and Fenland Research Ethics Committee for SLE sample and the Royal Free Hospital & Medical School Research Ethics Committee for T1D and HD samples.

Note that full information on the approval of the study protocol must also be provided in the manuscript.

Flow Cytometry

Plots

Confirm that:

- [x] The axis labels state the marker and fluorochrome used (e.g. CD4-FITC).
- [x] The axis scales are clearly visible. Include numbers along axes only for bottom left plot of group (a 'group' is an analysis of identical markers).
- [x] All plots are contour plots with outliers or pseudocolor plots.
- [x] A numerical value for number of cells or percentage (with statistics) is provided.

Methodology

Sample preparation	Peripheral blood mononuclear cells (PBMCs) were isolated from blood cones obtained from healthy donors. Where stated in text and figure legends cells were sorted using BD FASC Aria or enriched using magnetic beads. As described in material and methods and results sections, for some of the experiments cells were cultured in vitro before the flow cytometry analysis.
Instrument	Data were collected using FACS Canto II or BD FACS Aria I or II cell sorter.
Software	FlowJo software was used for data analysis.
Cell population abundance	Post-sort cell populations were generally > 95% pure as determined by flow cytometry.
Gating strategy	For all flow cytometry experiments the initial gating included FSS-A/SCC-A gating to gate leukocytes and FSS-A/FSS-H to exclude doublets and debris. Gates were set based on FMO (fluorescence minus one) for surface markers or on isotype staining for intracellular markers. Gating on proliferating cells was done based on VPD dilution.

- [x] Tick this box to confirm that a figure exemplifying the gating strategy is provided in the Supplementary Information.
